# Critical impact of automobile industry with advanced decision support system and Aczél-Alsina Hammy mean operators

**DOI:** 10.1038/s41598-025-24344-6

**Published:** 2026-01-08

**Authors:** Abrar Hussain, Kifayat Ullah, Zeeshan Ali, Dragan Pamucar

**Affiliations:** 1 Department of Mathematics, Riphah International University (Lahore Campus), Lahore, 54000 Pakistan; 2 Department of Mathematics, Saveetha School of Engineering, Saveetha Institute of Medical and Technical Sciences, Saveetha University, Chennai, Tamil Nadu 602105 India; 3 Department of Information Management, National Yunlin University of Science and Technology, 123 University Road, Section 3, Douliou, 64002 Yunlin Taiwan, R.O.C.; 4 Széchenyi István University, Győr, Hungary; 5 College of Mechatronics and Control Engineering , Shenzhen University, Shenzhen, 518060 China; 6 College of Computer Science and Software Engineering, Shenzhen University, Shenzhen, 518060 China

**Keywords:** Spherical fuzzy values, Aczel Alsina operations, Hamy mean and dual Hamy man models, Automobile industry and multi-attribute group decision-making process, Engineering, Mathematics and computing

## Abstract

The automobile industry plays a pivotal role in global economic development, technological innovation, and sustainable mobility solutions. It drives advancements in engineering, manufacturing, and smart technologies and influences transportation systems. A decision analysis system in the automobile industry serves as a structured framework to evaluate complex choices involving design, production, supply chain, marketing, and sustainability strategies based on vague human information. To achieve the main goal of this article, we explore the concepts of spherical fuzzy sets (SFSs) for handling uncertainty and vagueness in human judgments. The SFS is a more efficient and broader fuzzy framework that has extensive information about an object. Besides the concepts discussed in the fuzzy framework, we also modify the theory of Hamy mean (HM) models under Aczel Alsina operations. By combining two different theories of Aczel Alsina operations and Hamy mean models, we derive a family of mathematical models, namely spherical fuzzy Aczel Alsina Hamy mean (SFAHM) and spherical fuzzy Aczel Alsina weighted Hamy mean (SFAWHM) operators. Moreover, another generalization of the Dual HM (DHM) models is modified in the form of spherical fuzzy Aczel Alsina DHM (SFADHM) and spherical fuzzy Aczel Alsina weighted DHM (SFAWDHM) operators. Some reliable and appropriate characteristics are also studied to demonstrate the flexibility of the proposed operators. An intelligent decision algorithm of the multi-attribute group decision-making (MAGDM) problem is discussed to resolve real-life applications and a group of expert’s opinions. To see the effectiveness and reliability of newly developed terminologies, we discussed a numerical example to choose desirable alternatives under an automobile industry system. The influence study is also presented here by setting numerous parametric values in the currently discussed methodologies. To showcase the validation and superiority of diagnosed mathematical models, we establish a comparative study to compare the results of invented approaches with the results of existing terminologies.

## Introduction

MAGDM is an essential component of current cognitive technologies and has seen significant recent development in the disciplines of management, marketing, and other professions. Its main procedure is ranking options and choosing the best options out of a group of options based on a set of attribute values. Therefore, the fundamental problem with all MAGDM methods is how to combine attribute values efficiently. On the other hand, decision maker’s assessments of alternatives are never accurate or clear due to the subjective character of human intellectuals in realistic choice situations. The handling of fuzzy data and uncertainty is crucial because they occur frequently in real-world situations. To address and explain the uncertainties in the data in terms of establishing their positive value (PV) and negative value (NV) in the form of an intuitionistic fuzzy (IF) set (IFS) given by Atanassov^1^. The IFS is the modification of the fuzzy set (FS) developed by Zadeh^2^. These theories have received a lot of helpfulness from numerous researchers in recent years and have been successfully applied to various real-world scenarios. Yager^3^ anticipated the theory of pythagorean FS (PyFS) by relaxing the condition of IFS, such as the sum of the squares of PV and NV must lie on a closed interval $\left[0, 1\right]$. Sometimes, PyFSs are unable to handle dubious and uncertain information when exceeding the sum of PV and NV from the closed interval $0$ to $1$. To overcome these situations, Yager^4^ extended the idea of PyFSs with the sum of the power of PV and NV that lies on a closed interval $\left[0, 1\right]$. The PyFSs and IFSs are limited in particular scenarios and face complexities when human opinions are in more than two directions. Cuong^5,6^ gave a robust idea of picture FSs (PFSs) with four characteristics, including PV, indeterminacy value (IV), NV, and refusal value. In PFSs, having a certain condition in which the sum of PV, IV and NV lies in a close interval $\left[\text{0,1}\right]$. Mahmood et al.^7^ extended the concepts of PFSs in certain environments, namely spherical FSs (SFSs), with the sum of the squares of PV, IV and NV lying on a $\left[0, 1\right]$. Numerous researchers utilized the concepts of PFSs and SFSs in different fields of research environments, as seen in references^8–10^. Table 1 also demonstrates a comprehensive overview about different fuzzy frameworks.

**Table 1:** Comprehensive overview of fuzzy frameworks.

Framework	Positive value	Indeterminacy value	Negative value	Refusal value	Mathematical shape
Fuzsy set (FS)^2^	✕	✕	✕	✕	$0 \le M\left( \xi \right) \le 1$
Intuitionistic fuzzy set (IFS)^1^	✓	✕	✓	✕	$0 \le M\left( \xi \right) + N\left( \xi \right) \le 1$
Pythagorean fuzzy set (PyFS)^3^	✓	✕	✓	✕	$0 \le M^{2} \left( \xi \right) + N^{2} \left( \xi \right) \le 1$
rung orthopair fuzzy set (q-ROFS)^4^	✓	✕	✓	✕	$0 \le M^{q} \left( \xi \right) + N^{q} \left( \xi \right) \le 1$
Picture fuzzy set (PFS)^5^	✓	✓	✓	✓	$0 \le M\left( \xi \right) + I\left( \xi \right) + N\left( \xi \right) \le 1$
Spherical fuzzy set (SFS)^7^	✓	✓	✓	✓	$0 \le M^{2} \left( \xi \right) + I^{2} \left( \xi \right) + N^{2} \left( \xi \right) \le 1$

Aggregation operators are a hot research topic; recently, numerous scientists have worked on several AOs in different fields of fuzzy environments. These models play a significant role in the aggregation process and handle unpredictable and dubious information during the aggregation of data under the system of MAGDM approaches. Some existing AOs in different fuzzy environments, like Xu^11^, modified concepts of arithmetic mean and geometric mean, algebraic sum, and algebraic product under IF information. Xu^12^ also anticipated some similarity measures to improve the aggregation process under the system of IFSs. Arora and Garg^13^ introduced new powerful models in the form of prioritized operators to study the preference of objects based on MAGDM approaches. Chen et al.^14^ presented some distance measures and invented a list of AOs based on intuitionistic hesitant FS under the multi-criteria decision-making process. Certain AOs were developed by exploring the prioritized Linguistic fuzzy system theory from Arora and Garg^13^. Garg^15^ initiated the relations of correlations among PyF information and defined certain criteria for the decision-making of information. Akram et al.^16^ introduced some new models for the aggregation process based on the PyF system. Some geometric AOs based on a complex interval-valued PyF system and an application of a green supplier chain process under multi-criteria decision-making making also studied by Ali et al.^17^. Mahmood et al.^18^ anticipated a list of AOs based on frank operations by utilizing the theory of interval-valued PFSs. Ahmed and Dai^19^ initiated a distinction between two different environments of rough sets under the theory of PFSs. Hussain et al.^20^ presented the concepts of the vendor management system and introduced a list of new methodologies based on complex PFSs. Jana et al.^21^ modified algebraic operations to construct Dombi aggregation operators taking into account picture fuzzy information and the decision analysis problem. Akram et al.^22^ developed Dombi mathematical models to integrate uncertainty in human judgments under consideration of complex spherical fuzzy situations. Ashraf et al.^23^ proposed some hybrid approaches of Dombi t-norm and t-conorm taking into account spherical fuzzy information. Fahmi et al.^24^ deduced some robust operators of a cubic fuzzy framework to integrate ambiguous information of human judgments. Hussain et al.^25^ explored different qualities of physical education with conflicting criteria and a fuzzy-based decision algorithm of the decision-making problem. Garg^26^ established an optimization technique for resolving expert’s judgments by applying Einstein aggregation operators of pythagorean fuzzy information. Gao et al.^27^ examined the ranking of preferences based on prioritized Hamacher aggregation operators taking into account a hesitant bipolar fuzzy framework. Wu and Wei^28^ constructed hybrid mathematical models of Hamacher aggregation operators to integrate the ambiguous information of expert’s judgments. Darko and Liang^29^ established an intelligent optimization technique of the EDAS method to determine a suitable ranking of preferences using Hamacher aggregation operators. Li et al.^30^ applied the optimization technique of the WASPAS method to assess the qualities of crowdsourcing, incorporating spherical fuzzy cubic information. Hussain et al.^31^ investigated a robust digital security system under expert’s judgment and applied Sugeno-Weber aggregation operators of an intuitionistic fuzzy framework. Imran et al.^32^ resolved a group of expert’s opinions to investigate a suitable optimal option using Aczel Alsina based Bonferroni mean aggregation operators under interval-valued intuitionistic fuzzy information. Asif et al.^33^ elaborated the concepts of the pythagorean fuzzy framework to derive hybrid Hamacher mathematical models. Fujita^34^ developed some integrating models for handling uncertainty in complicated real-life applications and numerical examples. Shit and Ghorai^35^ applied the theory of the VIKOR method to determine a reliable charging method of public charging station in city. Shit and Ghorai^36^ scrutinized a brand of educational institute under the system of hesitant fuzzy framework and decision-making models. Shit and Ghorai^37^ developed reliable Dombi aggregation operators of Fermatean fuzzy context and decision analysis problem. Shit et al.^38^ also developed some Harmonic mean aggregation operators incorporating trapezoidal picture fuzzy situation and advanced decision support system.

Aczel Alsina explored the theory of triangular norms in the form of Aczel Alsina triangular norms. Aczel Alsina triangular norms are robust tools utilized to overcome the loss of information during the aggregation of data. Recently, Aczel Alsina models gained a lot of attention from numerous scholars in different fuzzy environments. Senapati et al.^39^ gave certain mathematical tools based on Aczel Alsina operations corresponding to the theory of IF information. Hussain et al.^40^ developed modified Frank aggregation operators to integrate a group of expert’s opinions, taking into account complex picture fuzzy information. Senapati et al.^41^ elaborated the theory of IFSs in the framework of interval-valued IFSs and initiated a list of certain models based on Aczel Alsina operations. Hussain et al.^42^ extended the concepts of Aczel Alsina operations in the system of PyFSs and gave certain models for multi-criteria decision-making techniques. Evaluate effective objects that are harmful to mango crops by utilizing Aczel Alsina operations under PFSs by Naeem et al.^43^. Hussain et al.^44^ explored basic properties of Muirhead mean operators with Frank aggregation operators and an intuitionistic fuzzy scenario. Naeem and Ali^45^ gave some robust mathematical tools to illustrate the given information by the experts under the system of the decision-making process.

Hara et al.^46^ introduced certain comprehensive methods to explore the interrelationship among crisp information in the form of HM and DHM operators. The HM and DHM operators are hot mathematical tools and play a key role in the field of research in different environments. Wu et al.^47^ elaborated on the tourism industry by using the discussed mathematical tools based on interval-valued IFSs (IVIFSs). Wu et al.^48^ extended the theory of Dombi operations under the system of IVIFSs and gave certain aggregation models to handle dubious and imprecise information. Liu and Liu^49^ utilized the theory of HM tools in the framework of linguistic HM tools and gave certain mathematical tools to solve real-life problems under MAGDM techniques. Li et al.^50^ anticipated a list of new aggregation models by using the theory of HM and DHM operators based on PyF information. Qin^51^ gave a new concept of interval 2-type fuzzy theory based on HM aggregation tools and established an application of the supplied system under the MAGDM approach. Liu and Wang^52^ improved traditional operational laws in the form of interactional operational laws and presented a series of new AOs based on IFSs. Wang et al.^53^ presented an enterprise resource management system and developed certain AOs of HM tools based on q-ROF information.

In the competitive and dynamic environment of the automobile industry, decision-making plays a critical role in maintaining market leadership and ensuring sustainable growth. Automakers face complex problems when evaluating potential suppliers, selecting materials, or choosing technological investments to enhance production efficiency and product quality. These decisions often involve conflicting criteria, such as cost, quality, environmental impact, and safety, requiring comprehensive assessments that consider objective data and expert opinions. Incorporating advanced decision-making tools and spherical fuzzy information allows decision-makers to handle uncertainty, imprecision, and varying degrees of positive, negative, and indeterminacy. This approach enhances the accuracy and reliability of selecting optimal solutions for supply chain management, product design, and strategic market positioning, leading to improved performance and competitive advantage.

### Motivation behind the proposed methodologies

The motivation behind developing the spherical fuzzy framework stems from the need to address complex decision-making scenarios where traditional fuzzy and intuitionistic fuzzy sets fall short in managing higher levels of uncertainty and imprecision. Spherical fuzzy sets extend the capabilities of picture fuzzy sets by incorporating three degrees—positive, negative, and indeterminacy—while ensuring that the sum of their squared values does not exceed one. This structure allows for a more flexible and comprehensive representation of uncertainty, accommodating situations where the exact levels of agreement and disagreement are difficult to determine with precision. The spherical fuzzy framework is particularly beneficial for real-world problems like supply chain management, risk analysis, and multi-criteria decision-making, where handling subjective judgments and uncertain data is critical. Its enhanced ability to capture vagueness and ambiguity empowers decision-makers to make more informed and robust decisions.

Incorporating Aczél-Alsina aggregation operators into spherical fuzzy environments is motivated by the need for sophisticated mathematical tools that preserve the nuances of spherical fuzzy information while effectively combining multiple criteria. Aczél-Alsina operators are known for their flexibility and ability to model varying degrees of compromise between criteria by adjusting parameter values, providing decision-makers with greater control over the aggregation process. These operators exhibit desirable properties, such as monotonicity and idempotency, which are critical for consistency in decision-making. By integrating Aczél-Alsina operators with spherical fuzzy logic, decision-makers can better manage conflicting criteria, non-linear preferences, and varying levels of risk tolerance. This approach enhances the precision and adaptability of aggregation processes, making it suitable for applications in complex industries like automotive manufacturing, healthcare, and financial analysis, where nuanced decision-making is essential for achieving optimal outcomes.

### Novelty and contributions

Keeping in mind the inspirations of HM models and Aczel Alsina operations, we discussed the primary contributions of the presentation as follows:

1. To fix uncertainty and vagueness in human judgments, expose the theoretical concepts of spherical fuzzy information with flexible operations and comparison rules. The SFS is a more convenient and efficient fuzzy framework that has extensive information about human judgments.
2. To modify the mathematical approaches of Hamy mean operators to demonstrate correlation among different conflicting criteria and key features.
3. Formulation of feasible operations of Aczel Alsina t-norm and t-conorm considering spherical fuzzy context.
4. Besides the concepts of spherical fuzzy information, we derive a family of hybrid mathematical approaches of Aczel Alsina HM operators, namely, SFAHM and SFAWHM operators. Moreover, a family of Dual HM operators based on Aczel Alsina operations is also derived, such as SFADHM and SFWDHM operators. To reveal the strength and validation of proposed models, we discussed the feasible properties of the discussed aggregation operators.
5. To highlight the validation and compatibility of proposed terminologies, we discussed the stepwise decision algorithm of the MAGDM problem with numerical examples related to the automobile industry.
6. Sensitivity analysis and comparison methods reveal the supremacy of derived mathematical approaches and decision-making models.

### Layout of the presentation

**Fig. 1:**
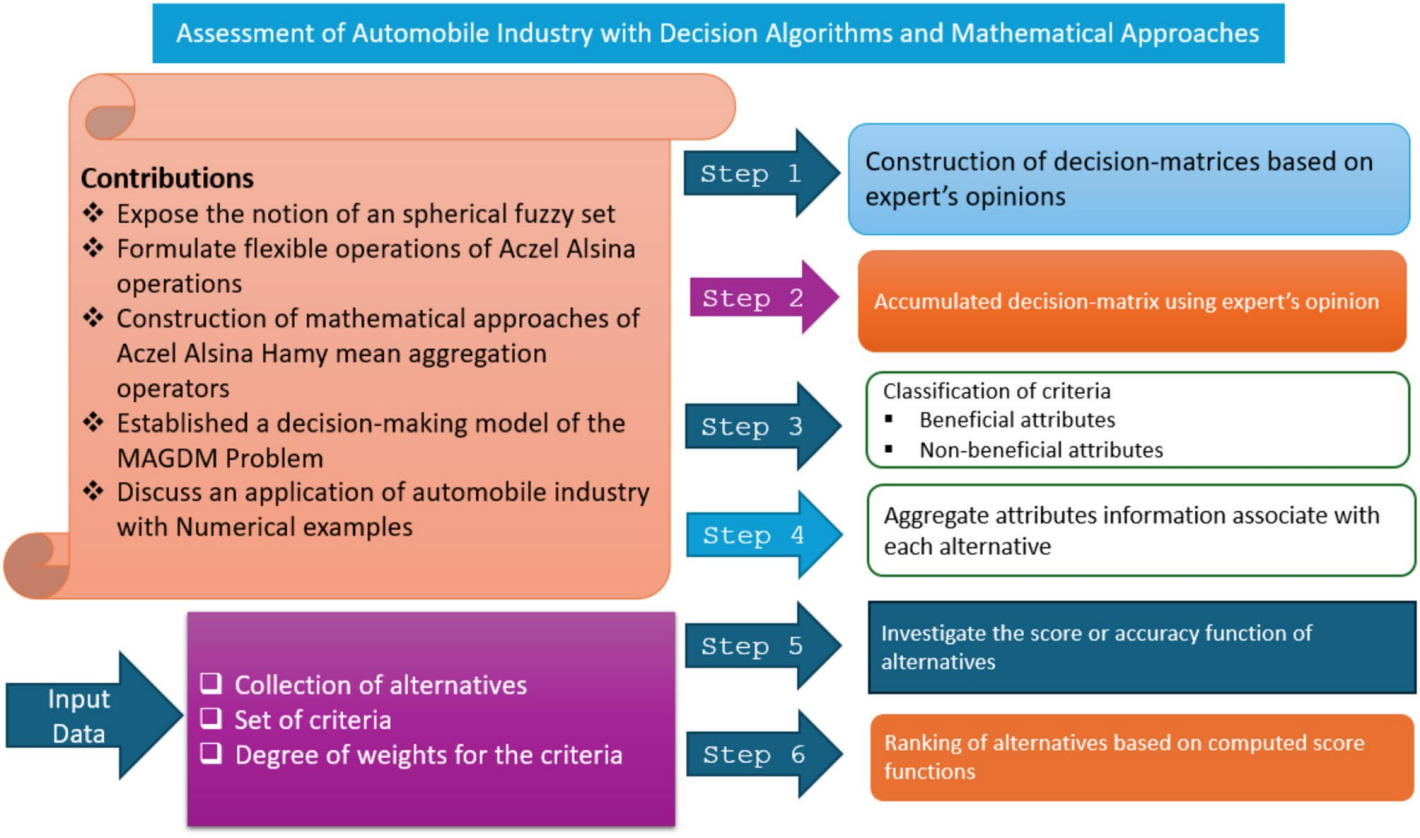
Explores primary contributions and the stepwise decision algorithm of the MAGDM problem.

The structure of this research document is maintained as follows: Section “Introduction” explores basic terminologies and further development of current research work. In Section “Preliminaries”, the notion of SFS, the rule of comparison for SF information and the tools of the interrelationship among fuzzy arguments are discussed. In Section “The proposed Aczel-Alsina Hammy mean aggregation operators of spherical fuzzy numbers”, we studied some robust operations of Aczel Alsina tools under the system of SF information. In Section “Spherical fuzzy Aczel-Alsina dual Hammy mean operators”, by utilizing certain tools of the HM operator, we presented a list of AOs, including SFAHM and SFAWHM operators based on Aczel Alsina operations, with some properties of the developed AOs. In Section “Decision algorithm for multi-attribute group decision-making problem”, we also elaborated on DHM operators in the form of SFADHM and SFAWDHM operators based on Aczel Alsina tools with desired properties. In Section “Influence study”, assessment of real-life problems under a certain decision-making tool of the MAGDM approach, we also developed a particular numerical example to select optimal alternatives under the system of the automobile industry. In Section “Comparatives study”, we determined the effectiveness of our proposed terminologies by comparing the results of our current approach with some existing approaches, and presented a summary of this article in Section “Conclusions”. Figure 1 demonstrates key contributions with a stepwise decision algorithm of the MAGDM problem.

## Preliminaries

In this section, we explore the idea of PFSs in the form of SFSs and their fundamental operations for further development of this article.

### List of symbols

**Table:** 

Symbols	Description	Symbols	Description
${\varvec{M}}$	Positive value	$I$	indeterminacy value
${\varvec{N}}$	Negative value	$X$	Universal set
${\varvec{\xi}}$	Element of the universal set	$\varphi$	Refusal value
${\varvec{\beta}}$	Spherical fuzzy value	$\lambda$	Scalar multiple
${\varvec{T}}$	Aczel Alsina t-norm	$S$	Aczel Alsina t-conorm
${\varvec{w}}$	Weight vector	$\beth$	Parametric variable of Aczel Alsina operations
${\varvec{\Gamma}}$	Alternative	${\varvec{\Lambda}}$	Attribute
$\mathbf{\rm K}$	Expert	$\Phi$	Weight of expert

#### Definition 1

A PFS $A$ on a universal set $X$ is defined as follows^6^:

$$A=\left\{\left(\xi , {M}_{A}\left(\xi \right), {I}_{A}\left(\xi \right), {N}_{A}\left(\xi \right)\right)|\xi \in X\right\}$$

Note that ${M}_{A}\in \left[\text{0,1}\right]$, ${I}_{A}\in \left[\text{0,1}\right]$ and ${N}_{A}\in \left[\text{0,1}\right]$ denote the positive value, indeterminacy value, and negative value of $\xi$, respectively. The mathematical condition of a PFS is given as:

$$0\le {M}_{A}\left(\xi \right)+{I}_{A}\left(\xi \right)+{N}_{A}\left(\xi \right)\le 1,$$

Moreover, the refusal value of $\xi$ is denoted by ${\varphi }_{A}\left(\xi \right)=1-\left({M}_{A}\left(\xi \right)+{I}_{A}\left(\xi \right)+{N}_{A}\left(\xi \right)\right)$.

#### Definition 2

Let $X$ be the universal set and SFS $A$ is shown as follows^7^:

$$A=\left\{\left(\xi , {M}_{A}\left(\xi \right), {I}_{A}\left(\xi \right), {N}_{A}\left(\xi \right)\right)|\xi \in X\right\}$$

where ${M}_{A}$, ${I}_{A}$ and ${N}_{A}$ denote the positive function, the indeterminacy function, and the negative function, respectively, ${M}_{A}:X\to \left[0, 1\right],$ ${I}_{A}:X\to \left[0, 1\right]$, ${N}_{A}:X\to \left[0, 1\right]$, ${M}_{A}(\xi )$, ${I}_{A}(\xi )$ and ${N}_{A}(\xi )$ denote the positive value (PV), the indeterminacy value (IV) and the negative value (NV) of $\xi$ in the SFS $A$, respectively, and $\xi \in X$. A SFS must satisfy the following conditions:

$$0\le {{M}_{A}}^{2}\left(\xi \right)+{{I}_{A}}^{2}\left(\xi \right)+{{N}_{A}}^{2}\left(\xi \right)\le 1,$$

where $\in X$. The refusal value ${\varphi }_{A}\left(\xi \right)$ of $\xi$ is denoted by ${\varphi }_{A}\left(\xi \right)=\sqrt{1-\left({{M}_{A}}^{2}\left(\xi \right)+{{I}_{A}}^{2}\left(\xi \right)+{{N}_{A}}^{2}\left(\xi \right)\right)}$, where $\xi$ $\in X$.

In a SFS $A=\left\{\left(\xi , {M}_{A}\left(\xi \right), {I}_{A}\left(\xi \right), {N}_{A}(\xi )\right)|\xi \in X\right\}$ and the triple $\beta =\left({M}_{A}\left(\xi \right), {I}_{A}\left(\xi \right), {N}_{A}\left(\xi \right)\right)$ is called a SFN^54^. For convenience, the representation of a SFN $\beta =\left({M}_{A}\left(\xi \right), {I}_{A}\left(\xi \right), {N}_{A}\left(\xi \right)\right)$ is abbreviated into $\beta =\left({M}_{A}, {I}_{A}, {N}_{A}\right)$.

#### Definition 3

Let ${\beta }_{1}=\left({M}_{1}, {I}_{1}, {N}_{1}\right)$ and ${\beta }_{2}=\left({M}_{2}, {I}_{2}, {N}_{2}\right)$ be two SFNs. Then^7^:

(i) $${\beta }_{1}\subseteq {\beta }_{2}\text{ iff}\;{M}_{1}\le {M}_{2}, {I}_{1}\le {I}_{2}$$ and $${N}_{1}\le {N}_{2}$$.
(ii) $${\beta }_{1}={\beta }_{2} \text{iff} {\beta }_{1}\subseteq {\beta }_{2}$$ and $${\beta }_{2}\subseteq {\beta }_{1}$$.
(iii) $${\beta }_{1}\cup {\beta }_{2}=\left(max\left({M}_{1}, {M}_{2}\right), min\left({I}_{1}, {I}_{2}\right), min\left({N}_{1}, {N}_{2}\right)\right)$$.
(iv) $${\beta }_{1}\cap {\beta }_{2}=\left(min\left({M}_{1}, {M}_{2}\right), min\left({I}_{1}, {I}_{2}\right), max\left({N}_{1}, {N}_{2}\right)\right)$$.
(v) $${\beta }_{1}^{c}=\left({N}_{1}, {I}_{1},{M}_{1}\right)$$.

#### Definition 4

Let ${\beta }_{1}=\left({M}_{1}, {I}_{1}, {N}_{1}\right)$ and ${\beta }_{2}=\left({M}_{2}, {I}_{2}, {N}_{2}\right)$ be two SFNs and let $\lambda \ge 0.$ Then^45^,

(i) $${\beta }_{1}\oplus {\beta }_{2}=\left(\sqrt{{{M}_{1}}^{2}+{{M}_{2}}^{2}-{{M}_{1}}^{2}\times {{M}_{2}}^{2}},{I}_{1}\times {I}_{2},{N}_{1}\times {N}_{2}\right)$$.
(ii) $${\beta }_{1}\otimes {\beta }_{2}=\left({M}_{1}.{M}_{2},{I}_{1}.{I}_{2}, \sqrt{{{N}_{1}}^{2}+{{N}_{2}}^{2}-{{N}_{1}}^{2}\times {{N}_{2}}^{2}}\right)$$.
(iiii) $$\lambda {\beta }_{1}=\left(\sqrt{1-{\left(1-{{M}_{1}}^{2}\right)}^{\lambda }, }{\left({I}_{1}\right)}^{\lambda }, {\left({N}_{1}\right)}^{\lambda }\right)$$.
(iv) $${{\beta }_{1}}^{\lambda }=\left({\left({M}_{1}\right)}^{\lambda },{\left({I}_{1}\right)}^{\lambda } , \sqrt{1-{\left(1-{\left({N}_{1}\right)}^{2}\right)}^{\lambda }}\right)$$.

#### Definition 5

Let $\beta =\left(M, I, N\right)$ be a SFN. The score value $S\left(\beta \right)$ and the accuracy value $A\left(\beta \right)$ of the SFN $\beta$ are defined as follows^10^:

1 $$S\left(\beta \right)=\frac{1}{3}\left(2+{M}^{2}- {I}^{2}- {N}^{2}\right)$$

2 $$A\left(\beta \right)=\frac{1}{3}\left({M}^{2}+ {I}^{2}+ {N}^{2}\right)$$

where $$S\left(\beta \right)$$ $$\in \left[0, 1\right]$$ and $$A\left(\beta \right)$$ $$\in \left[0, 1\right]$$.

#### Definition 6

Let ${\beta }_{1}=\left({M}_{1}, {I}_{1}, {N}_{1}\right)$, ${\beta }_{2}=\left({M}_{2}, {I}_{2}, {N}_{2}\right)$, …, and ${\beta }_{n}=\left({M}_{n}, {I}_{n}, {N}_{n}\right)$ be SFNs. The SF weighted averaging operator (SFWA) of the SFNs ${\beta }_{1}$, ${\beta }_{2}$, …, and ${\beta }_{n}$ is shown as follows^7^:

$$SFWA\left( {\beta_{1} ,\beta_{2} ,{ } \ldots ,\beta_{n} } \right) = \mathop \sum \limits_{\varsigma = 1}^{n} { }\omega_{\varsigma } { }\beta_{\varsigma } ,$$

where $\omega_{\varsigma }$ denotes the weight of the SFN $\beta_{\varsigma }$, $\omega_{\varsigma } \in \left[ {0, 1} \right]$,$\varsigma = 1,2, \ldots ,n$, and $\mathop \sum \limits_{\varsigma = 1}^{n} \omega_{\varsigma } = 1.$

#### Definition 7

Let ${K}_{1}$, ${K}_{2}$, …, and ${K}_{n}$ be any real numbers. The HM operator $H{M}^{\left(\nu \right)}$ of the real numbers ${K}_{1}$, ${K}_{2}$, …, and ${K}_{n}$ is shown as follows^46^:

3 $$HM^{\left( \nu \right)} \left( {K_{1} ,K_{2} , \ldots ,K_{n} } \right) = \frac{{\mathop \sum \nolimits_{{1 \le \varsigma_{1} < , \ldots , < \varsigma_{\nu } \le n}} \left( {\mathop \prod \nolimits_{j = 1}^{\nu } K_{{\varsigma_{j} }} } \right)^{{\frac{1}{\nu }}} }}{{C_{n}^{\nu } }},$$

where $1\le \nu \le n$ and ${C}_{n}^{\nu }=\frac{n!}{\nu !\left(n-\nu \right)!}$. The HM operator $H{M}^{\left(\nu \right)}$ has the following properties:

(i) If $${K}_{1}={K}_{2}=\cdots ={K}_{n}=\beta$$, then $$H{M}^{\left(\nu \right)}\left({\beta }_{1},{\beta }_{2},\cdots ,{\beta }_{k}\right)=\beta$$.
(ii) $HM^{\left( \nu \right)} \left( {K_{1} ,K_{2} , \ldots ,K_{\varsigma } } \right) \le HM^{\left( \nu \right)} \left( {Q_{1} ,Q_{2} , \ldots ,Q_{\varsigma } } \right)$ if $K_{\varsigma } \le Q_{\varsigma }$, where $Q_{\varsigma }$ is a real number and $\varsigma = 1, 2, \ldots ,n$.
(iii) $$\min \left( {K_{\varsigma } } \right) \le HM^{\left( \nu \right)} \left( {K_{1} ,K_{2} , \ldots ,K_{\varsigma } } \right) \le maxK_{\varsigma }$$, where $$\varsigma = 1, 2, \ldots ,n$$.
(iv) The arithmetic mean operator $HM^{\left( \nu \right)} \left( {K_{1} ,K_{2} , \ldots ,K_{\varsigma } } \right) = \frac{1}{\varsigma }\mathop \sum \limits_{\varsigma = 1}^{n} K_{\varsigma }$.
(v) The geometric mean operator $HM^{\left( \nu \right)} \left( {K_{1} ,K_{2} , \ldots ,K_{\varsigma } } \right) = \left( {\mathop \prod \limits_{\varsigma = 1}^{k} K_{\varsigma } } \right)^{{\frac{1}{\nu }}}$.

In the following, we review the definition of the Dual Hammy mean operator (DHM)^46^ operator.

#### Definition 8

Let ${K}_{1}$, ${K}_{2}$, …, and ${K}_{n}$ be any real numbers. DHM operator $DH{M}^{\left(\nu \right)}$ of the real numbers ${K}_{1}$, ${K}_{2}$, …, and ${K}_{n}$ is shown as follows^46^:

4 $$DHM^{\left( \nu \right)} \left( {K_{1} ,K_{2} , \ldots ,K_{n} } \right) = \left( {\mathop \prod \limits_{{1 \le \varsigma_{1} < , \ldots , < \varsigma_{\nu } \le n}} \left( {\frac{{\mathop \sum \nolimits_{j = 1}^{\nu } K_{{\varsigma_{j} }} }}{\nu }} \right)} \right)^{{\frac{1}{{C_{n}^{\nu } }}}} ,$$

where $$1\le \nu \le n$$ and $${C}_{n}^{\nu }=\frac{n!}{\nu !\left(n-\nu \right)!}$$.

#### Definition 9

The mathematical theory of the Aczel Alsina t-norm and t-conorm is given by^55^:

$${T}^{\beth }\left(\alpha , \beta \right)={e}^{-{\left({\left(-ln\alpha \right)}^{\beth }+{\left(-ln\beta \right)}^{\beth }\right)}^{\frac{1}{\beth }}}$$

and

$${S}^{\beth }\left(\alpha , \beta \right)=1-{e}^{-{\left({\left(-ln\alpha \right)}^{\beth }+{\left(-ln\beta \right)}^{\beth }\right)}^{\frac{1}{\beth }}}$$

where $$1\le \beth \le +\infty$$.

#### Definition 10

Let ${\beta }_{1}=\left({M}_{1}, {I}_{1}, {N}_{1}\right)$, ${\beta }_{2}=\left({M}_{2}, {I}_{2}, {N}_{2}\right)$ and $\beta =\left(M, I, N\right)$ be SFNs, let $\lambda >0$ and let $\beth >1$. The Aczel-Alsina operations of SFNs are discussed as follows:

(i) $$\beta_{1} \oplus \beta_{2} = \left( \begin{gathered} \sqrt {1 - e^{{ - \left( {\left( { - \ln \left( {1 - \left( {M_{1} } \right)^{2} } \right)} \right)^{{\beth }} + \left( { - \ln \left( {1 - \left( {M_{2} } \right)^{2} } \right)} \right)^{{\beth }} } \right)^{{\frac{1}{{\beth }}}} }} } , \hfill \\ e^{{ - \left( {\left( { - \ln \left( {I_{1} } \right)} \right)^{{\beth }} + \left( { - \ln \left( {I_{2} } \right)} \right)^{{\beth }} } \right)^{{\frac{1}{{\beth }}}} }} , \hfill \\ e^{{ - \left( {\left( { - \ln \left( {N_{1} } \right)} \right)^{{\beth }} + \left( { - \ln \left( {N_{2} } \right)} \right)^{{\beth }} } \right)^{{\frac{1}{{\beth }}}} }} \hfill \\ \end{gathered} \right)$$
(ii) $$\beta_{1} \otimes \beta_{2} = \left( \begin{gathered} e^{{ - \left( {\left( { - \ln \left( {M_{1} } \right)} \right)^{{\beth }} + \left( { - \ln \left( {M_{2} } \right)} \right)^{{\beth }} } \right)^{{\frac{1}{{\beth }}}} }} , \hfill \\ e^{{ - \left( {\left( { - \ln \left( {I_{1} } \right)} \right)^{{\beth }} + \left( { - \ln \left( {I_{2} } \right)} \right)^{{\beth }} } \right)^{{\frac{1}{{\beth }}}} }} , \hfill \\ \sqrt {1 - e^{{ - \left( {\left( { - \ln \left( {1 - \left( {N_{1} } \right)^{2} } \right)} \right)^{{\beth }} + \left( { - \ln \left( {1 - \left( {N_{2} } \right)^{2} } \right)} \right)^{{\beth }} } \right)^{{\frac{1}{{\beth }}}} }} } \hfill \\ \end{gathered} \right)$$
(iii) $$\lambda \beta = \left( \begin{gathered} \sqrt {1 - e^{{ - \left( {\lambda \left( { - \ln \left( {1 - \left( M \right)^{2} } \right)} \right)^{{\beth }} } \right)^{{\frac{1}{{\beth }}}} }} } , \hfill \\ e^{{ - \left( {\lambda \left( { - \ln \left( I \right)} \right)^{{\beth }} } \right)^{{\frac{1}{{\beth }}}} }} , \hfill \\ e^{{ - \left( {\lambda \left( { - \ln \left( N \right)} \right)^{{\beth }} } \right)^{{\frac{1}{{\beth }}}} }} \hfill \\ \end{gathered} \right)$$
(iv) $$\beta^{\lambda } = \left( \begin{gathered} e^{{ - \left( {\lambda \left( { - \ln \left( M \right)} \right)^{{\beth }} } \right)^{{\frac{1}{{\beth }}}} }} , \hfill \\ e^{{ - \left( {\lambda \left( { - \ln \left( I \right)} \right)^{{\beth }} } \right)^{{\frac{1}{{\beth }}}} }} , \hfill \\ \sqrt {1 - e^{{ - \left( {\lambda \left( { - \ln \left( {1 - \left( N \right)^{2} } \right)} \right)^{{\beth }} } \right)^{{\frac{1}{{\beth }}}} }} } \hfill \\ \end{gathered} \right)$$

## The proposed Aczel-Alsina Hammy mean aggregation operators of spherical fuzzy numbers

In this section, we propose the spherical fuzzy Aczel-Alsina Hammy mean (SFAAHM) operator and the spherical fuzzy Aczel-Alsina weighted Hammy mean (SFAAWHM) operator of spherical fuzzy numbers (SFNs).

### Definition 11

Let ${\beta }_{1}=\left({M}_{1}, {I}_{1}, {N}_{1}\right)$, ${\beta }_{2}=\left({M}_{2}, {I}_{2}, {N}_{2}\right)$, …, and ${\beta }_{n}=\left({M}_{n}, {I}_{n}, {N}_{n}\right)$ be SFNs. The proposed SFAAHM operator of the SFNs ${\beta }_{1}$, ${\beta }_{2}$, …, and ${\beta }_{n}$ is defined as follows:

5 $$SFAAHM^{\left( \nu \right)} \left( {\beta_{1} ,\beta_{2} , \ldots ,\beta_{n} } \right) = \frac{{ \oplus_{{1 \le \varsigma_{1} < , \ldots , < \varsigma_{\nu } \le n}} \left( { \otimes_{j = 1}^{\nu } {\upbeta }_{{\varsigma_{j} }} } \right)^{{\frac{1}{\nu }}} }}{{C_{n}^{\nu } }}$$

where $$1\le \nu \le n$$ and $${C}_{n}^{\nu }=\frac{n!}{\nu !\left(n-\nu \right)!}$$.

### Theorem 1

Let ${\beta }_{1}=\left({M}_{1}, {I}_{1}, {N}_{1}\right)$, ${\beta }_{2}=\left({M}_{2}, {I}_{2}, {N}_{2}\right)$, …, and ${\beta }_{n}=\left({M}_{n}, {I}_{n}, {N}_{n}\right)$ be SFNs. The aggregated value of the SFAAHM operator is a SFN, shown as follows:

6 $$\begin{gathered} SFAAHM^{{\left( \nu \right)}} \left( {\beta _{1} ,\beta _{2} , \ldots ,\beta _{n} } \right) \hfill \\ \quad = \left( \begin{gathered} \sqrt {1 - e^{{ - \left( {\left( {\frac{1}{{C_{n}^{\nu } }}} \right)\left( {\mathop \sum \limits_{{1 \le \varsigma _{1} < ,~ \ldots ,~ < \varsigma _{\nu } \le n}} \left( { - \ln \left( {1 - \left( {e^{{ - \left( {\left( {\frac{1}{\nu }} \right)\left( {\mathop \sum \limits_{{j = 1}}^{\nu } \left( { - \ln \left( {M_{{\varsigma _{j} }} } \right)} \right)^{\beth } } \right)} \right)^{{\frac{1}{\beth }}} }} } \right)^{2} } \right)} \right)^{\beth } } \right)} \right)^{{\frac{1}{\beth }}} }} } , \hfill \\ e^{{ - \left( {\left( {\frac{1}{{C_{n}^{\nu } }}} \right)\left( {\mathop \sum \limits_{{1 \le \varsigma _{1} < ,~ \ldots ,~ < \varsigma _{\nu } \le n}} \left( {\left( {\frac{1}{\nu }} \right)\left( {\mathop \sum \limits_{{j = 1}}^{\nu } \left( { - \ln \left( {I_{{\varsigma _{j} }} } \right)} \right)^{\beth } } \right)} \right)} \right)} \right)^{{\frac{1}{\beth }}} }} , \hfill \\ e^{{ - \left( {\left( {\frac{1}{{C_{n}^{\nu } }}} \right)\left( {\mathop \sum \limits_{{1 \le \varsigma _{1} < ,~ \ldots ,~ < \varsigma _{\nu } \le n}} \left( { - \ln \left( {\sqrt {1 - e^{{ - \left( {\left( {\frac{1}{\nu }} \right)\left( {\mathop \sum \limits_{{j = 1}}^{\nu } \left( { - \ln \left( {1 - \left( {N_{{\varsigma _{j} }} } \right)^{2} } \right)} \right)^{\beth } } \right)} \right)^{{\frac{1}{\beth }}} }} } } \right)} \right)^{\beth } } \right)} \right)^{{\frac{1}{\beth }}} }} \hfill \\ \end{gathered} \right) \hfill \\ \end{gathered}$$

### Proof

Because ${\beta }_{1}=\left({M}_{1}, {I}_{1}, {N}_{1}\right)$, ${\beta }_{2}=\left({M}_{2}, {I}_{2}, {N}_{2}\right)$, …, and ${\beta }_{n}=\left({M}_{n}, {I}_{n}, {N}_{n}\right)$ are SFNs, we get:

$$\mathop {\mathop \otimes \limits_{j = 1} }\limits^{\nu } {\upbeta }_{{\varsigma_{j} }} = \left( \begin{gathered} e^{{ - \left( {\mathop \sum \limits_{j = 1}^{\nu } \left( { - \ln \left( {M_{{\varsigma_{j} }} } \right)} \right)^{\beth } } \right)^{{\frac{1}{\beth }}} }} , \hfill \\ e^{{ - \left( {\mathop \sum \limits_{j = 1}^{\nu } \left( { - \ln \left( {I_{{\varsigma_{j} }} } \right)} \right)^{\beth } } \right)^{{\frac{1}{\beth }}} }} , \hfill \\ \sqrt {1 - e^{{ - \left( {\mathop \sum \limits_{j = 1}^{\nu } \left( { - \ln \left( {1 - \left( {N_{{\varsigma_{j} }} } \right)^{2} } \right)} \right)^{\beth } } \right)^{{\frac{1}{\beth }}} }} } \hfill \\ \end{gathered} \right)$$

$$\left( {\mathop {\mathop \otimes \limits_{j = 1} }\limits^{\nu } {\upbeta }_{{\varsigma_{j} }} } \right)^{{\frac{1}{\nu }}} = \left( \begin{gathered} e^{{ - \left( {\left( {\frac{1}{\nu }} \right)\left( {\mathop \sum \limits_{j = 1}^{\nu } \left( { - \ln \left( {M_{{\varsigma_{j} }} } \right)} \right)^{\beth } } \right)} \right)^{{\frac{1}{\beth }}} }} , \hfill \\ e^{{ - \left( {\left( {\frac{1}{\nu }} \right)\left( {\mathop \sum \limits_{j = 1}^{\nu } \left( { - \ln \left( {I_{{\varsigma_{j} }} } \right)} \right)^{\beth } } \right)} \right)^{{\frac{1}{\beth }}} }} , \hfill \\ \sqrt {1 - e^{{ - \left( {\left( {\frac{1}{\nu }} \right)\left( {\mathop \sum \limits_{j = 1}^{\nu } \left( { - \ln \left( {1 - \left( {N_{{\varsigma_{j} }} } \right)^{2} } \right)} \right)^{\beth } } \right)} \right)^{{\frac{1}{\beth }}} }} } \hfill \\ \end{gathered} \right)$$

$$\mathop \oplus \limits_{{1 \le \varsigma_{1} < , \ldots , < \varsigma_{\nu } \le n}} \left( {\mathop {\mathop \otimes \limits_{j = 1} }\limits^{\nu } {\upbeta }_{{\varsigma_{j} }} } \right)^{{\frac{1}{\nu }}} = \left( \begin{gathered} \sqrt {1 - e^{{ - \left( {\mathop \sum \limits_{{1 \le \varsigma_{1} < , \ldots , < \varsigma_{\nu } \le n}} \left( { - \ln \left( {1 - \left( {e^{{ - \left( {\left( {\frac{1}{\nu }} \right)\left( {\mathop \sum \limits_{j = 1}^{\nu } \left( { - \ln \left( {M_{{\varsigma_{j} }} } \right)} \right)^{\beth } } \right)} \right)^{{\frac{1}{\beth }}} }} } \right)^{2} } \right)} \right)^{\beth } } \right)^{{\frac{1}{\beth }}} }} } , \hfill \\ e^{{ - \left( {\mathop \sum \limits_{{1 \le \varsigma_{1} < , \ldots , < \varsigma_{\nu } \le n}} \left( {\left( {\frac{1}{\nu }} \right)\left( {\mathop \sum \limits_{j = 1}^{\nu } \left( { - \ln \left( {I_{{\varsigma_{j} }} } \right)} \right)^{\beth } } \right)} \right)} \right)^{{\frac{1}{\beth }}} }} , \hfill \\ e^{{ - \left( {\mathop \sum \limits_{{1 \le \varsigma_{1} < , \ldots , < \varsigma_{\nu } \le n}} \left( { - \ln \left( {\sqrt {1 - e^{{ - \left( {\left( {\frac{1}{\nu }} \right)\left( {\mathop \sum \limits_{j = 1}^{\nu } \left( { - \ln \left( {1 - \left( {N_{{\varsigma_{j} }} } \right)^{2} } \right)} \right)^{\beth } } \right)} \right)^{{\frac{1}{\beth }}} }} } } \right)} \right)^{\beth } } \right)^{{\frac{1}{\beth }}} }} \hfill \\ \end{gathered} \right)$$

$$\frac{{\mathop \oplus \limits_{{1 \le \varsigma_{1} < , \ldots , < \varsigma_{\nu } \le n}} \left( {\mathop {\mathop \otimes \limits_{j = 1} }\limits^{\nu } {\upbeta }_{{\varsigma_{j} }} } \right)^{{\frac{1}{\nu }}} }}{{C_{n}^{\nu } }} = \left( \begin{gathered} \sqrt {1 - e^{{ - \left( {\left( {\frac{1}{{C_{n}^{\nu } }}} \right)\left( {\mathop \sum \limits_{{1 \le \varsigma_{1} < , \ldots , < \varsigma_{\nu } \le n}} \left( { - \ln \left( {1 - \left( {e^{{ - \left( {\left( {\frac{1}{\nu }} \right)\left( {\mathop \sum \limits_{j = 1}^{\nu } \left( { - \ln \left( {M_{{\varsigma_{j} }} } \right)} \right)^{\beth } } \right)} \right)^{{\frac{1}{\beth }}} }} } \right)^{2} } \right)} \right)^{\beth } } \right)} \right)^{{\frac{1}{\beth }}} }} } , \hfill \\ e^{{ - \left( {\left( {\frac{1}{{C_{n}^{\nu } }}} \right)\left( {\mathop \sum \limits_{{1 \le \varsigma_{1} < , \ldots , < \varsigma_{\nu } \le n}} \left( {\left( {\frac{1}{\nu }} \right)\left( {\mathop \sum \limits_{j = 1}^{\nu } \left( { - \ln \left( {I_{{\varsigma_{j} }} } \right)} \right)^{\beth } } \right)} \right)} \right)} \right)^{{\frac{1}{\beth }}} }} , \hfill \\ e^{{ - \left( {\left( {\frac{1}{{C_{n}^{\nu } }}} \right)\left( {\mathop \sum \limits_{{1 \le \varsigma_{1} < , \ldots , < \varsigma_{\nu } \le n}} \left( { - \ln \left( {\sqrt {1 - e^{{ - \left( {\left( {\frac{1}{\nu }} \right)\left( {\mathop \sum \limits_{j = 1}^{\nu } \left( { - \ln \left( {1 - \left( {N_{{\varsigma_{j} }} } \right)^{2} } \right)} \right)^{\beth } } \right)} \right)^{{\frac{1}{\beth }}} }} } } \right)} \right)^{\beth } } \right)} \right)^{{\frac{1}{\beth }}} }} \hfill \\ \end{gathered} \right)$$

### Theorem 2

Let ${\beta }_{1}=\left({M}_{1}, {I}_{1}, {N}_{1}\right)$, ${\beta }_{2}=\left({M}_{2}, {I}_{2}, {N}_{2}\right)$, …, and ${\beta }_{n}=\left({M}_{n}, {I}_{n}, {N}_{n}\right)$ be identical SFNs, where ${\beta }_{1}={\beta }_{2}=$ $\cdots = {\beta }_{n}=\beta =( M, I, N$). Then, ${SFAAHM}^{\left(\nu \right)}\left({\beta }_{1},{\beta }_{2},\cdots ,{\beta }_{n}\right)= \beta$.

### Proof

Because $$\beta _{1} = \beta _{2} =$$$$\cdots = \beta _{n} = \beta$$$$= \left( {M,I,N} \right)$$, we get $${M}_{1}={M}_{2}=\cdots = {M}_{n}=M$$, $${I}_{1}={I}_{2}=\cdots = {I}_{n}=I$$, and $$N_{1} = N_{2} =$$$$\cdots$$$$= N_{n} = N$$. Based on Eq. (6), we get

$$SFAAHM^{\left( \nu \right)} \left( {\beta_{1} ,\beta_{2} , \ldots ,\beta_{n} } \right) = \left( \begin{gathered} \sqrt {1 - e^{{ - \left( {\left( {\frac{1}{{C_{n}^{\nu } }}} \right)\left( {\mathop \sum \limits_{{1 \le \varsigma_{1} < , \ldots , < \varsigma_{\nu } \le n}} \left( { - \ln \left( {1 - \left( {e^{{ - \left( {\left( {\frac{1}{\nu }} \right)\left( {\mathop \sum \limits_{j = 1}^{\nu } \left( { - \ln \left( {M_{{\varsigma_{j} }} } \right)} \right)^{\beth } } \right)} \right)^{{\frac{1}{\beth }}} }} } \right)^{2} } \right)} \right)^{\beth } } \right)} \right)^{{\frac{1}{\beth }}} }} } , \hfill \\ e^{{ - \left( {\left( {\frac{1}{{C_{n}^{\nu } }}} \right)\left( {\mathop \sum \limits_{{1 \le \varsigma_{1} < , \ldots , < \varsigma_{\nu } \le n}} \left( {\left( {\frac{1}{\nu }} \right)\left( {\mathop \sum \limits_{j = 1}^{\nu } \left( { - \ln \left( {I_{{\varsigma_{j} }} } \right)} \right)^{\beth } } \right)} \right)} \right)} \right)^{{\frac{1}{\beth }}} }} , \hfill \\ e^{{ - \left( {\left( {\frac{1}{{C_{n}^{\nu } }}} \right)\left( {\mathop \sum \limits_{{1 \le \varsigma_{1} < , \ldots , < \varsigma_{\nu } \le n}} \left( { - \ln \left( {\sqrt {1 - e^{{ - \left( {\left( {\frac{1}{\nu }} \right)\left( {\mathop \sum \limits_{j = 1}^{\nu } \left( { - \ln \left( {1 - \left( {N_{{\varsigma_{j} }} } \right)^{2} } \right)} \right)^{\beth } } \right)} \right)^{{\frac{1}{\beth }}} }} } } \right)} \right)^{\beth } } \right)} \right)^{{\frac{1}{\beth }}} }} \hfill \\ \end{gathered} \right)$$

$$= \left( \begin{gathered} \sqrt {1 - e^{{ - \left( {\left( {\frac{1}{{C_{n}^{\nu } }}} \right)\left( {\mathop \sum \limits_{{1 \le \varsigma_{1} < , \ldots , < \varsigma_{\nu } \le n}} \left( { - \ln \left( {1 - \left( {e^{{ - \left( {\left( {\frac{1}{\nu }} \right)\left( { - \ln \left( {M_{j} } \right)} \right)^{\beth } } \right)^{{\frac{1}{\beth }}} }} } \right)^{2} } \right)} \right)^{\beth } } \right)} \right)^{{\frac{1}{\beth }}} }} } , \hfill \\ e^{{ - \left( {\left( {\frac{1}{{C_{n}^{\nu } }}} \right)\left( {\mathop \sum \limits_{{1 \le \varsigma_{1} < , \ldots , < \varsigma_{\nu } \le n}} \left( {\left( {\frac{1}{\nu }} \right)\left( { - \ln \left( {I_{j} } \right)} \right)^{\beth } } \right)} \right)} \right)^{{\frac{1}{\beth }}} }} , \hfill \\ e^{{ - \left( {\left( {\frac{1}{{C_{n}^{\nu } }}} \right)\left( {\mathop \sum \limits_{{1 \le \varsigma_{1} < , \ldots , < \varsigma_{\nu } \le n}} \left( { - \ln \left( {\sqrt {1 - e^{{ - \left( {\left( {\frac{1}{\nu }} \right)\left( { - \ln \left( {1 - \left( {N_{j} } \right)^{2} } \right)} \right)^{\beth } } \right)^{{\frac{1}{\beth }}} }} } } \right)} \right)^{\beth } } \right)} \right)^{{\frac{1}{\beth }}} }} \hfill \\ \end{gathered} \right)$$

$$= \left( \begin{gathered} \sqrt {1 - e^{{ - \left( {\left( { - \ln \left( {1 - \left( {e^{{ - \left( {\left( {\frac{1}{\nu }} \right)\left( { - \ln \left( {M_{j} } \right)} \right)^{\beth } } \right)^{{\frac{1}{\beth }}} }} } \right)^{2} } \right)} \right)^{\beth } } \right)^{{\frac{1}{\beth }}} }} } , \hfill \\ e^{{ - \left( {\left( {\left( {\frac{1}{\nu }} \right)\left( { - \ln \left( {I_{j} } \right)} \right)^{\beth } } \right)} \right)^{{\frac{1}{\beth }}} }} , \hfill \\ e^{{ - \left( {\left( { - \ln \left( {\sqrt {1 - e^{{ - \left( {\left( {\frac{1}{\nu }} \right)\left( { - \ln \left( {1 - \left( {N_{j} } \right)^{2} } \right)} \right)^{\beth } } \right)^{{\frac{1}{\beth }}} }} } } \right)} \right)^{\beth } } \right)^{{\frac{1}{\beth }}} }} \hfill \\ \end{gathered} \right) = \beta$$

### Theorem 3

Let $\beta_{\varsigma } = \left( {M_{\varsigma } , I_{\varsigma } , N_{\varsigma } } \right)$ and $\beta_{\varsigma }^{\prime} = \left( {M_{\varsigma }^{\prime} , I_{\varsigma }^{\prime} , N_{\varsigma }^{\prime} } \right)$ be SFNs, where $\varsigma = 1, 2, \ldots ,n$. If $\beta_{\varsigma } \le \beta_{\varsigma }^{\prime}$, where $\varsigma = 1, 2, \ldots ,n$, then

$${SFAAHM}^{\left(\nu \right)}\left({\beta }_{1},{\beta }_{2},\ldots ,{\beta }_{n}\right)\le {SFAAHM}^{\left(\nu \right)}\left({\beta }_{1}^{\prime},{\beta }_{2}^{\prime},\ldots ,{\beta }_{n}^{\prime}\right).$$

### Proof

Because $\beta_{\varsigma } \le \beta_{\varsigma }^{\prime}$, where $\beta_{\varsigma } = \left( {M_{\varsigma } , I_{\varsigma } , N_{\varsigma } } \right)$, $\beta_{\varsigma }^{\prime} = \left( {M_{\varsigma }^{\prime} , I_{\varsigma }^{\prime} , N_{\varsigma }^{\prime} } \right)$ and $\varsigma = 1, 2, \ldots ,n$, we get

$$\begin{gathered} \sqrt {1 - e^{{ - \left( {\left( {\frac{1}{{C_{n}^{\nu } }}} \right)\left( {\mathop \sum \limits_{{1 \le \varsigma_{1} < , \ldots , < \varsigma_{\nu } \le n}} \left( { - \ln \left( {1 - \left( {e^{{ - \left( {\left( {\frac{1}{\nu }} \right)\left( {\mathop \sum \limits_{j = 1}^{\nu } \left( { - \ln \left( {M_{{\varsigma_{j} }} } \right)} \right)^{\beth } } \right)} \right)^{{\frac{1}{\beth }}} }} } \right)^{2} } \right)} \right)^{\beth } } \right)} \right)^{{\frac{1}{\beth }}} }} } \hfill \\ \quad \le \sqrt {1 - e^{{ - \left( {\left( {\frac{1}{{C_{n}^{\nu } }}} \right)\left( {\mathop \sum \limits_{{1 \le \varsigma_{1} < , \ldots , < \varsigma_{\nu } \le n}} \left( { - \ln \left( {1 - \left( {e^{{ - \left( {\left( {\frac{1}{\nu }} \right)\left( {\mathop \sum \limits_{j = 1}^{\nu } \left( { - \ln \left( {M_{{\varsigma_{j} }}^{\prime} } \right)} \right)^{\beth } } \right)} \right)^{{\frac{1}{\beth }}} }} } \right)^{2} } \right)} \right)^{\beth } } \right)} \right)^{{\frac{1}{\beth }}} }} } \hfill \\ \end{gathered}$$

Then, we get

$$\begin{gathered} e^{{ - \left( {\left( {\frac{1}{{C_{n}^{\nu } }}} \right)\left( {\mathop \sum \limits_{{1 \le \varsigma_{1} < , \ldots , < \varsigma_{\nu } \le n}} \left( {\left( {\frac{1}{\nu }} \right)\left( {\mathop \sum \limits_{j = 1}^{\nu } \left( { - \ln \left( {I_{{\varsigma_{j} }} } \right)} \right)^{\beth } } \right)} \right)} \right)} \right)^{{\frac{1}{\beth }}} }} \hfill \\ \quad \ge e^{{ - \left( {\left( {\frac{1}{{C_{n}^{\nu } }}} \right)\left( {\mathop \sum \limits_{{1 \le \varsigma_{1} < , \ldots , < \varsigma_{\nu } \le n}} \left( {\left( {\frac{1}{\nu }} \right)\left( {\mathop \sum \limits_{j = 1}^{\nu } \left( { - \ln \left( {I_{{\varsigma_{j} }}^{\prime} } \right)} \right)^{\beth } } \right)} \right)} \right)} \right)^{{\frac{1}{\beth }}} }} \hfill \\ \end{gathered}$$

and

$$\begin{gathered} e^{{ - \left( {\left( {\frac{1}{{C_{n}^{\nu } }}} \right)\left( {\mathop \sum \limits_{{1 \le \varsigma_{1} < , \ldots , < \varsigma_{\nu } \le n}} \left( { - \ln \left( {\sqrt {1 - e^{{ - \left( {\left( {\frac{1}{\nu }} \right)\left( {\mathop \sum \limits_{j = 1}^{\nu } \left( { - \ln \left( {1 - \left( {N_{{\varsigma_{j} }} } \right)^{2} } \right)} \right)^{\beth } } \right)} \right)^{{\frac{1}{\beth }}} }} } } \right)} \right)^{\beth } } \right)} \right)^{{\frac{1}{\beth }}} }} \hfill \\ \quad \ge e^{{ - \left( {\left( {\frac{1}{{C_{n}^{\nu } }}} \right)\left( {\mathop \sum \limits_{{1 \le \varsigma_{1} < , \ldots , < \varsigma_{\nu } \le n}} \left( { - \ln \left( {\sqrt {1 - e^{{ - \left( {\left( {\frac{1}{\nu }} \right)\left( {\mathop \sum \limits_{j = 1}^{\nu } \left( { - \ln \left( {1 - \left( {N_{{\varsigma_{j} }}^{\prime} } \right)^{2} } \right)} \right)^{\beth } } \right)} \right)^{{\frac{1}{\beth }}} }} } } \right)} \right)^{\beth } } \right)} \right)^{{\frac{1}{\beth }}} }} \hfill \\ \end{gathered}$$

Therefore, we get:

$${SFAAHM}^{\left(\nu \right)}\left({\beta }_{1},{\beta }_{2},\ldots ,{\beta }_{n}\right)\le {SFAAHM}^{\left(\nu \right)}\left({\beta }_{1}^{\prime},{\beta }_{2}^{\prime},\ldots ,{\beta }_{n}^{\prime}\right).$$

### Theorem 4

Let ${\beta }_{1}=\left({M}_{1}, {I}_{1}, {N}_{1}\right)$, ${\beta }_{2}=\left({M}_{2}, {I}_{2}, {N}_{2}\right)$, …, and ${\beta }_{n}=\left({M}_{n}, {I}_{n}, {N}_{n}\right)$ be SFNs. If $\beta ^{ - } = \min \left( {\beta _{1} ,\beta _{2} , \ldots ,\beta _{n} } \right)$ and $\beta ^{ + } = \max \left( {\beta _{1} ,\beta _{2} , \ldots ,\beta _{n} } \right)$. Then,

$${\beta }^{-}\le SFAAWA\left({\beta }_{1}, {\beta }_{2}, \ldots , {\beta }_{n}\right)\le {\beta }^{+}.$$

### Proof

Let $$\beta ^{ - } = \min \left( {\beta _{1} ,\beta _{2} , \ldots ,\beta _{n} } \right)$$$$= \left( {M_{\varsigma }^{ - } ,I_{\varsigma }^{ - } ,N_{\varsigma }^{ - } } \right)$$ and let $$\beta ^{ + } = \max \left( {\beta _{1} ,\beta _{2} , \ldots ,\beta _{n} } \right) )$$$$= \left( {M_{\varsigma }^{ + } ,I_{\varsigma }^{ + } ,N_{\varsigma }^{ + } } \right)$$, where $$M^{ - } = \min _{\varsigma } \left\{ {M_{\varsigma } } \right\},$$$$I^{ - } = \max _{\varsigma } \left\{ {I_{\varsigma } } \right\},$$$$N^{ - } = \max _{\varsigma } \left\{ {N_{\varsigma } } \right\}$$, $$M^{ + } = \max _{\varsigma } \left\{ {M_{\varsigma } } \right\},$$$$I^{ + } = \min _{\varsigma } \left\{ {I_{\varsigma } } \right\}$$ and $$N^{ + } = \min_{\varsigma } \left\{ {N_{\varsigma } } \right\}$$.

Hence, we get:

$$\left( \begin{gathered} \sqrt {1 - e^{{ - \left( {\left( {\frac{1}{{C_{n}^{\nu } }}} \right)\left( {\mathop \sum \limits_{{1 \le \varsigma_{1} < , \ldots , < \varsigma_{\nu } \le n}} \left( { - \ln \left( {1 - \left( {e^{{ - \left( {\left( {\frac{1}{\nu }} \right)\left( {\mathop \sum \limits_{j = 1}^{\nu } \left( { - \ln \left( {M_{{\varsigma_{j} }} } \right)} \right)^{\beth } } \right)} \right)^{{\frac{1}{\beth }}} }} } \right)^{2} } \right)} \right)^{\beth } } \right)} \right)^{{\frac{1}{\beth }}} }} } , \hfill \\ e^{{ - \left( {\left( {\frac{1}{{C_{n}^{\nu } }}} \right)\left( {\mathop \sum \limits_{{1 \le \varsigma_{1} < , \ldots , < \varsigma_{\nu } \le n}} \left( {\left( {\frac{1}{\nu }} \right)\left( {\mathop \sum \limits_{j = 1}^{\nu } \left( { - \ln \left( {I_{{\varsigma_{j} }} } \right)} \right)^{\beth } } \right)} \right)} \right)} \right)^{{\frac{1}{\beth }}} }} , \hfill \\ e^{{ - \left( {\left( {\frac{1}{{C_{n}^{\nu } }}} \right)\left( {\mathop \sum \limits_{{1 \le \varsigma_{1} < , \ldots , < \varsigma_{\nu } \le n}} \left( { - \ln \left( {\sqrt {1 - e^{{ - \left( {\left( {\frac{1}{\nu }} \right)\left( {\mathop \sum \limits_{j = 1}^{\nu } \left( { - \ln \left( {1 - \left( {N_{{\varsigma_{j} }} } \right)^{2} } \right)} \right)^{\beth } } \right)} \right)^{{\frac{1}{\beth }}} }} } } \right)} \right)^{\beth } } \right)} \right)^{{\frac{1}{\beth }}} }} \hfill \\ \end{gathered} \right)$$

$$\begin{gathered} \sqrt {1 - e^{{ - \left( {\left( {\frac{1}{{C_{n}^{\nu } }}} \right)\left( {\mathop \sum \limits_{{1 \le \varsigma_{1} < , \ldots , < \varsigma_{\nu } \le n}} \left( { - \ln \left( {1 - \left( {e^{{ - \left( {\left( {\frac{1}{\nu }} \right)\left( {\mathop \sum \limits_{j = 1}^{\nu } \left( { - \ln \left( {M^{ - } } \right)} \right)^{\beth } } \right)} \right)^{{\frac{1}{\beth }}} }} } \right)^{2} } \right)} \right)^{\beth } } \right)} \right)^{{\frac{1}{\beth }}} }} } \hfill \\ \quad \le \sqrt {1 - e^{{ - \left( {\left( {\frac{1}{{C_{n}^{\nu } }}} \right)\left( {\mathop \sum \limits_{{1 \le \varsigma_{1} < , \ldots , < \varsigma_{\nu } \le n}} \left( { - \ln \left( {1 - \left( {e^{{ - \left( {\left( {\frac{1}{\nu }} \right)\left( {\mathop \sum \limits_{j = 1}^{\nu } \left( { - \ln \left( {M_{{\varsigma_{j} }} } \right)} \right)^{\beth } } \right)} \right)^{{\frac{1}{\beth }}} }} } \right)^{2} } \right)} \right)^{\beth } } \right)} \right)^{{\frac{1}{\beth }}} }} } \hfill \\ \quad \le \sqrt {1 - e^{{ - \left( {\left( {\frac{1}{{C_{n}^{\nu } }}} \right)\left( {\mathop \sum \limits_{{1 \le \varsigma_{1} < , \ldots , < \varsigma_{\nu } \le n}} \left( { - \ln \left( {1 - \left( {e^{{ - \left( {\left( {\frac{1}{\nu }} \right)\left( {\mathop \sum \limits_{j = 1}^{\nu } \left( { - \ln \left( {M^{ + } } \right)} \right)^{\beth } } \right)} \right)^{{\frac{1}{\beth }}} }} } \right)^{2} } \right)} \right)^{\beth } } \right)} \right)^{{\frac{1}{\beth }}} }} } \hfill \\ \end{gathered}$$

and

$$\begin{gathered} e^{{ - \left( {\left( {\frac{1}{{C_{n}^{\nu } }}} \right)\left( {\mathop \sum \limits_{{1 \le \varsigma_{1} < , \ldots , < \varsigma_{\nu } \le n}} \left( {\left( {\frac{1}{\nu }} \right)\left( {\mathop \sum \limits_{j = 1}^{\nu } \left( { - \ln \left( {I^{ - } } \right)} \right)^{\beth } } \right)} \right)} \right)} \right)^{{\frac{1}{\beth }}} }} \hfill \\ \quad \le e^{{ - \left( {\left( {\frac{1}{{C_{n}^{\nu } }}} \right)\left( {\mathop \sum \limits_{{1 \le \varsigma_{1} < , \ldots , < \varsigma_{\nu } \le n}} \left( {\left( {\frac{1}{\nu }} \right)\left( {\mathop \sum \limits_{j = 1}^{\nu } \left( { - \ln \left( {I_{{\varsigma_{j} }} } \right)} \right)^{\beth } } \right)} \right)} \right)} \right)^{{\frac{1}{\beth }}} }} \hfill \\ \quad \le e^{{ - \left( {\left( {\frac{1}{{C_{n}^{\nu } }}} \right)\left( {\mathop \sum \limits_{{1 \le \varsigma_{1} < , \ldots , < \varsigma_{\nu } \le n}} \left( {\left( {\frac{1}{\nu }} \right)\left( {\mathop \sum \limits_{j = 1}^{\nu } \left( { - \ln \left( {I^{ + } } \right)} \right)^{\beth } } \right)} \right)} \right)} \right)^{{\frac{1}{\beth }}} }} \hfill \\ \end{gathered}$$

and

$$\begin{gathered} e^{{ - \left( {\left( {\frac{1}{{C_{n}^{\nu } }}} \right)\left( {\mathop \sum \limits_{{1 \le \varsigma_{1} < , \ldots , < \varsigma_{\nu } \le n}} \left( { - \ln \left( {\sqrt {1 - e^{{ - \left( {\left( {\frac{1}{\nu }} \right)\left( {\mathop \sum \limits_{j = 1}^{\nu } \left( { - \ln \left( {1 - \left( {N^{ - } } \right)^{2} } \right)} \right)^{\beth } } \right)} \right)^{{\frac{1}{\beth }}} }} } } \right)} \right)^{\beth } } \right)} \right)^{{\frac{1}{\beth }}} }} \hfill \\ \quad \le e^{{ - \left( {\left( {\frac{1}{{C_{n}^{\nu } }}} \right)\left( {\mathop \sum \limits_{{1 \le \varsigma_{1} < , \ldots , < \varsigma_{\nu } \le n}} \left( { - \ln \left( {\sqrt {1 - e^{{ - \left( {\left( {\frac{1}{\nu }} \right)\left( {\mathop \sum \limits_{j = 1}^{\nu } \left( { - \ln \left( {1 - \left( {N_{{\varsigma_{j} }} } \right)^{2} } \right)} \right)^{\beth } } \right)} \right)^{{\frac{1}{\beth }}} }} } } \right)} \right)^{\beth } } \right)} \right)^{{\frac{1}{\beth }}} }} \hfill \\ \quad \le e^{{ - \left( {\left( {\frac{1}{{C_{n}^{\nu } }}} \right)\left( {\mathop \sum \limits_{{1 \le \varsigma_{1} < , \ldots , < \varsigma \le n}} \left( { - \ln \left( {\sqrt {1 - e^{{ - \left( {\left( {\frac{1}{\nu }} \right)\left( {\mathop \sum \limits_{j = 1}^{\nu } \left( { - \ln \left( {1 - \left( {N^{ + } } \right)^{2} } \right)} \right)^{\beth } } \right)} \right)^{{\frac{1}{\beth }}} }} } } \right)} \right)^{\beth } } \right)} \right)^{{\frac{1}{\beth }}} }} . \hfill \\ \end{gathered}$$

Therefore, we get

$${\beta }^{-}\le SFAAWA\left({\beta }_{1}, {\beta }_{2}, \ldots , {\beta }_{n}\right)\le {\beta }^{+}.$$

### Definition 12

Let ${\beta }_{1}=\left({M}_{1}, {I}_{1}, {N}_{1}\right)$, ${\beta }_{2}=\left({M}_{2}, {I}_{2}, {N}_{2}\right)$, …, and ${\beta }_{n}=\left({M}_{n}, {I}_{n}, {N}_{n}\right)$ be SFNs. The proposed SFAAWHM operator ${SFAAWHM}^{\left(\nu \right)}$ of the SFNs ${\beta }_{1}$, ${\beta }_{2}$, …, and ${\beta }_{n}$ is defined as follows:

7 $$SFAAWHM^{\left( \nu \right)} \left( {\beta_{1} ,\beta_{2} , \ldots ,\beta_{n} } \right) = \left\{ \begin{gathered} \frac{{ \oplus_{{1 \le \varsigma_{1} < , \ldots , < \varsigma_{\nu } \le n}} \left( {1 - \mathop \sum \nolimits_{j = 1}^{\nu } w_{j} } \right)\left( {\mathop {\mathop \otimes \limits_{j = 1} }\limits^{\nu } \beta_{{\varsigma_{j} }} } \right)^{{\frac{1}{\nu }}} }}{{C_{n - 1}^{\nu } }},\quad \quad {\text{if}}\;1 \le \nu < n \hfill \\ \otimes_{j = 1}^{\nu } \beta_{{\varsigma_{j} }}^{{\frac{{1 - w_{\varsigma } }}{n - 1}}} ,\quad \quad \quad \quad \quad \quad \quad \quad \quad \quad \quad \quad \quad \quad \quad \quad \;\;{\text{if}}\;\nu = n \hfill \\ \end{gathered} \right.$$

where $w_{j}$ be the weight vectors of $\beta_{\varsigma }$ such that $w_{j} \in \left[ {0, 1} \right]$ and $\sum\nolimits_{j = 1}^{n} {w_{j} = 1}$.

### Theorem 5

Let ${\beta }_{1}=\left({M}_{1}, {I}_{1}, {N}_{1}\right)$, ${\beta }_{2}=\left({M}_{2}, {I}_{2}, {N}_{2}\right)$, …, and ${\beta }_{n}=\left({M}_{n}, {I}_{n}, {N}_{n}\right)$ be SFNs. The aggregated result ${SFAAWHM}^{\left(\nu \right)}\left({\beta }_{1},{\beta }_{2},\ldots ,{\beta }_{n}\right)$ of the SFNs ${\beta }_{1}$, ${\beta }_{2}$, …, and ${\beta }_{n}$ is still a SFN, where

8 $$\begin{gathered} SFAAWHM^{\left( \nu \right)} \left( {\beta_{1} ,\beta_{2} , \ldots ,\beta_{n} } \right) \hfill \\ \quad = \left\{ \begin{gathered} \left( \begin{gathered} \sqrt {1 - e^{{ - \left( {\left( {\frac{1}{{C_{n - 1}^{\nu } }}} \right)\left( {\mathop \sum \limits_{{1 \le \varsigma_{1} < , \ldots , < \varsigma_{\nu } \le n}} \left( {\left( {1 - \mathop \sum \limits_{j = 1}^{\nu } w_{j} } \right)\left( { - \ln \left( {1 - \left( {e^{{ - \left( {\left( {\frac{1}{\nu }} \right)\left( {\mathop \sum \limits_{j = 1}^{\nu } \left( { - \ln \left( {M_{{\varsigma_{j} }} } \right)} \right)^{\beth } } \right)} \right)^{{\frac{1}{\beth }}} }} } \right)^{2} } \right)} \right)^{\beth } } \right)} \right)} \right)^{{\frac{1}{\beth }}} }} } , \hfill \\ e^{{ - \left( {\left( {\frac{1}{{C_{n - 1}^{\nu } }}} \right)\left( {\mathop \sum \limits_{{1 \le \varsigma_{1} < , \ldots , < \varsigma_{\nu } \le n}} \left( { - \ln \left( {e^{{ - \left( {\left( {1 - \mathop \sum \limits_{j = 1}^{\nu } w_{j} } \right)\left( {\left( {\frac{1}{\nu }} \right)\left( {\mathop \sum \limits_{j = 1}^{\nu } \left( { - \ln \left( {I_{{\varsigma_{j} }} } \right)} \right)^{\beth } } \right)} \right)} \right)^{{\frac{1}{\beth }}} }} } \right)} \right)^{\beth } } \right)} \right)^{{\frac{1}{\beth }}} }} , \hfill \\ e^{{ - \left( {\left( {\frac{1}{{C_{n - 1}^{\nu } }}} \right)\left( {\mathop \sum \limits_{{1 \le \varsigma_{1} < , \ldots , < \varsigma_{\nu } \le n}} \left( {\left( {1 - \mathop \sum \limits_{j = 1}^{\nu } w_{j} } \right)\left( { - \ln \left( {\sqrt {1 - e^{{ - \left( {\left( {\frac{1}{\nu }} \right)\left( {\mathop \sum \limits_{j = 1}^{\nu } \left( { - \ln \left( {1 - \left( {N_{{\varsigma_{j} }} } \right)^{2} } \right)} \right)^{\beth } } \right)} \right)^{{\frac{1}{\beth }}} }} } } \right)} \right)^{\beth } } \right)} \right)} \right)^{{\frac{1}{\beth }}} }} \hfill \\ \end{gathered} \right)\quad \;{\text{if}}\;1 \le \nu < n \hfill \\ \mathop {\mathop \otimes \limits_{j = 1} }\limits^{\nu } \beta_{{\varsigma_{j} }}^{{\frac{{1 - w_{j} }}{n - 1}}} = \left( \begin{gathered} e^{{ - \left( {\mathop \sum \limits_{j = 1}^{\nu } \left( {\left( {\left( {\frac{{1 - w_{j} }}{n - 1}} \right)\left( { - \ln \left( {M_{{\varsigma_{j} }} } \right)} \right)^{\beth } } \right)} \right)} \right)^{{\frac{1}{\beth }}} }} , \hfill \\ e^{{ - \left( {\mathop \sum \limits_{j = 1}^{\nu } \left( {\left( {\left( {\frac{{1 - w_{j} }}{n - 1}} \right)\left( { - \ln \left( {I_{{\varsigma_{j} }} } \right)} \right)^{\beth } } \right)} \right)} \right)^{{\frac{1}{\beth }}} }} , \hfill \\ \sqrt {1 - e^{{ - \left( {\mathop \sum \limits_{j = 1}^{\nu } \left( {\left( {\frac{{1 - w_{j} }}{n - 1}} \right)\left( { - \ln \left( {1 - \left( {N_{{\varsigma_{j} }} } \right)^{2} } \right)} \right)^{\beth } } \right)} \right)^{{\frac{1}{\beth }}} }} } \hfill \\ \end{gathered} \right)\quad \quad \quad \quad \quad \quad \quad \quad \quad \quad \quad \quad \quad \quad \quad \;{\text{if}}\;\nu = n \hfill \\ \end{gathered} \right. \hfill \\ \end{gathered}$$

where $$\beth >1$$.

### Proof

Because ${\beta }_{1}=\left({M}_{1}, {I}_{1}, {N}_{1}\right)$, ${\beta }_{2}=\left({M}_{2}, {I}_{2}, {N}_{2}\right)$, …, and ${\beta }_{n}=\left({M}_{n}, {I}_{n}, {N}_{n}\right)$ be SFNs, we prove Eq. (8) by using Definition 9.

*Case 1*: If $1\le \nu <n$, then:

$$\mathop {\mathop \otimes \limits_{j = 1} }\limits^{\nu } {\upbeta }_{{\varsigma_{j} }} = \left( \begin{gathered} e^{{ - \left( {\mathop \sum \limits_{j = 1}^{\nu } \left( { - \ln \left( {M_{{\varsigma_{j} }} } \right)} \right)^{\beth } } \right)^{{\frac{1}{\beth }}} }} , \hfill \\ e^{{ - \left( {\mathop \sum \limits_{j = 1}^{\nu } \left( { - \ln \left( {I_{{\varsigma_{j} }} } \right)} \right)^{\beth } } \right)^{{\frac{1}{\beth }}} }} , \hfill \\ \sqrt {1 - e^{{ - \left( {\mathop \sum \limits_{j = 1}^{\nu } \left( { - \ln \left( {1 - \left( {N_{{\varsigma_{j} }} } \right)^{2} } \right)} \right)^{\beth } } \right)^{{\frac{1}{\beth }}} }} } \hfill \\ \end{gathered} \right)$$

$$\left( {\mathop {\mathop \otimes \limits_{j = 1} }\limits^{\nu } {\upbeta }_{{\varsigma_{j} }} } \right)^{{\frac{1}{\nu }}} = \left( \begin{gathered} e^{{ - \left( {\left( {\frac{1}{\nu }} \right)\left( {\mathop \sum \limits_{j = 1}^{\nu } \left( { - \ln \left( {M_{{\varsigma_{j} }} } \right)} \right)^{\beth } } \right)} \right)^{{\frac{1}{\beth }}} }} , \hfill \\ e^{{ - \left( {\left( {\frac{1}{\nu }} \right)\left( {\mathop \sum \limits_{j = 1}^{\nu } \left( { - \ln \left( {I_{{\varsigma_{j} }} } \right)} \right)^{\beth } } \right)} \right)^{{\frac{1}{\beth }}} }} , \hfill \\ \sqrt {1 - e^{{ - \left( {\left( {\frac{1}{\nu }} \right)\left( {\mathop \sum \limits_{j = 1}^{\nu } \left( { - \ln \left( {1 - \left( {N_{{\varsigma_{j} }} } \right)^{2} } \right)} \right)^{\beth } } \right)} \right)^{{\frac{1}{\beth }}} }} } \hfill \\ \end{gathered} \right)$$

$$\begin{gathered} \left( {1 - \mathop \sum \limits_{j = 1}^{\nu } w_{j} } \right)\left( {\mathop {\mathop \otimes \limits_{j = 1} }\limits^{\nu } {\upbeta }_{{\varsigma_{j} }} } \right)^{{\frac{1}{\nu }}} \hfill \\ \quad = \left( \begin{gathered} \sqrt {1 - e^{{ - \left( {\left( {1 - \mathop \sum \limits_{j = 1}^{\nu } w_{j} } \right)\left( { - \ln \left( {1 - \left( {e^{{ - \left( {\left( {\frac{1}{\nu }} \right)\left( {\mathop \sum \limits_{j = 1}^{\nu } \left( { - \ln \left( {M_{{\varsigma_{j} }} } \right)} \right)^{\beth } } \right)} \right)^{{\frac{1}{\beth }}} }} } \right)^{2} } \right)} \right)^{\beth } } \right)^{{\frac{1}{\beth }}} }} } , \hfill \\ e^{{ - \left( {\left( {1 - \mathop \sum \limits_{j = 1}^{\nu } w_{j} } \right)\left( {\left( {\frac{1}{\nu }} \right)\left( {\mathop \sum \limits_{j = 1}^{\nu } \left( { - \ln \left( {I_{{\varsigma_{j} }} } \right)} \right)^{\beth } } \right)} \right)} \right)^{{\frac{1}{\beth }}} }} , \hfill \\ e^{{ - \left( {\left( {1 - \mathop \sum \limits_{j = 1}^{\nu } w_{j} } \right)\left( { - \ln \left( {\sqrt {1 - e^{{ - \left( {\left( {\frac{1}{\nu }} \right)\left( {\mathop \sum \limits_{j = 1}^{\nu } \left( { - \ln \left( {1 - \left( {N_{{\varsigma_{j} }} } \right)^{2} } \right)} \right)^{\beth } } \right)} \right)^{{\frac{1}{\beth }}} }} } } \right)} \right)^{\beth } } \right)^{{\frac{1}{\beth }}} }} \hfill \\ \end{gathered} \right) \hfill \\ \end{gathered}$$

$$\begin{gathered} \mathop \oplus \limits_{{1 \le \varsigma_{1} < , \ldots , < \varsigma_{\nu } \le n}} \left( {1 - \mathop \sum \limits_{j = 1}^{\nu } w_{{\varsigma_{j} }} } \right)\left( {\mathop {\mathop \otimes \limits_{j = 1} }\limits^{\nu } {\upbeta }_{{\varsigma_{j} }} } \right)^{{\frac{1}{\nu }}} \hfill \\ \quad = \left( \begin{gathered} \sqrt {1 - e^{{ - \left( {\mathop \sum \limits_{{1 \le \varsigma_{1} < , \ldots , < \varsigma_{\nu } \le n}} \left( {\left( {1 - \mathop \sum \limits_{j = 1}^{\nu } w_{j} } \right)\left( { - \ln \left( {1 - \left( {e^{{ - \left( {\left( {\frac{1}{\nu }} \right)\left( {\mathop \sum \limits_{j = 1}^{\nu } \left( { - \ln \left( {M_{{\varsigma_{j} }} } \right)} \right)^{\beth } } \right)} \right)^{{\frac{1}{\beth }}} }} } \right)^{2} } \right)} \right)^{\beth } } \right)} \right)^{{\frac{1}{\beth }}} }} } , \hfill \\ e^{{ - \left( {\mathop \sum \limits_{{1 \le \varsigma_{1} < , \ldots , < \varsigma_{\nu } \le n}} \left( { - \ln \left( {e^{{ - \left( {\left( {1 - \mathop \sum \limits_{j = 1}^{\nu } w_{j} } \right)\left( {\left( {\frac{1}{\nu }} \right)\left( {\mathop \sum \limits_{j = 1}^{\nu } \left( { - \ln \left( {I_{{\varsigma_{j} }} } \right)} \right)^{\beth } } \right)} \right)} \right)^{{\frac{1}{\beth }}} }} } \right)} \right)^{\beth } } \right)^{{\frac{1}{\beth }}} }} , \hfill \\ e^{{ - \left( {\mathop \sum \limits_{{1 \le \varsigma_{1} < , \ldots , < \varsigma_{\nu } \le n}} \left( {\left( {1 - \mathop \sum \limits_{j = 1}^{\nu } w_{j} } \right)\left( { - \ln \left( {\sqrt {1 - e^{{ - \left( {\left( {\frac{1}{\nu }} \right)\left( {\mathop \sum \limits_{j = 1}^{\nu } \left( { - \ln \left( {1 - \left( {N_{{\varsigma_{j} }} } \right)^{2} } \right)} \right)^{\beth } } \right)} \right)^{{\frac{1}{\beth }}} }} } } \right)} \right)^{\beth } } \right)} \right)^{{\frac{1}{\beth }}} }} \hfill \\ \end{gathered} \right) \hfill \\ \end{gathered}$$

$$\begin{gathered} \frac{{\mathop \oplus \limits_{{1 \le \varsigma_{1} < , \ldots , < \varsigma_{\nu } \le n}} \left( {1 - \mathop \sum \nolimits_{j = 1}^{\nu } w_{{\varsigma_{j} }} } \right)\left( {\mathop {\mathop \otimes \limits_{j = 1} }\limits^{\nu } {\upbeta }_{{\varsigma_{j} }} } \right)^{{\frac{1}{\nu }}} }}{{C_{n - 1}^{\nu } }} \hfill \\ \quad = \left( \begin{gathered} \sqrt {1 - e^{{ - \left( {\left( {\frac{1}{{C_{n - 1}^{\nu } }}} \right)\left( {\mathop \sum \limits_{{1 \le \varsigma_{1} < , \ldots , < \varsigma_{\nu } \le n}} \left( {\left( {1 - \mathop \sum \limits_{j = 1}^{\nu } w_{j} } \right)\left( { - \ln \left( {1 - \left( {e^{{ - \left( {\left( {\frac{1}{\nu }} \right)\left( {\mathop \sum \limits_{j = 1}^{\nu } \left( { - \ln \left( {M_{{\varsigma_{j} }} } \right)} \right)^{\beth } } \right)} \right)^{{\frac{1}{\beth }}} }} } \right)^{2} } \right)} \right)^{\beth } } \right)} \right)} \right)^{{\frac{1}{\beth }}} }} } , \hfill \\ e^{{ - \left( {\left( {\frac{1}{{C_{n - 1}^{\nu } }}} \right)\left( {\mathop \sum \limits_{{1 \le \varsigma_{1} < , \ldots , < \varsigma_{\nu } \le n}} \left( { - \ln \left( {e^{{ - \left( {\left( {1 - \mathop \sum \limits_{j = 1}^{\nu } w_{j} } \right)\left( {\left( {\frac{1}{\nu }} \right)\left( {\mathop \sum \limits_{j = 1}^{\nu } \left( { - \ln \left( {I_{{\varsigma_{j} }} } \right)} \right)^{\beth } } \right)} \right)} \right)^{{\frac{1}{\beth }}} }} } \right)} \right)^{\beth } } \right)} \right)^{{\frac{1}{\beth }}} }} , \hfill \\ e^{{ - \left( {\left( {\frac{1}{{C_{n - 1}^{\nu } }}} \right)\left( {\mathop \sum \limits_{{1 \le \varsigma_{1} < , \ldots , < \varsigma_{\nu } \le n}} \left( {\left( {1 - \mathop \sum \limits_{j = 1}^{\nu } w_{j} } \right)\left( { - \ln \left( {\sqrt {1 - e^{{ - \left( {\left( {\frac{1}{\nu }} \right)\left( {\mathop \sum \limits_{j = 1}^{\nu } \left( { - \ln \left( {1 - \left( {N_{{\varsigma_{j} }} } \right)^{2} } \right)} \right)^{\beth } } \right)} \right)^{{\frac{1}{\beth }}} }} } } \right)} \right)^{\beth } } \right)} \right)} \right)^{{\frac{1}{\beth }}} }} \hfill \\ \end{gathered} \right) \hfill \\ \end{gathered}$$

*Case 2*: If $\nu =n$, then we get

$${\upbeta }_{{\varsigma_{j} }}^{{\frac{{1 - w_{j} }}{n - 1}}} = \left( \begin{gathered} e^{{ - \left( {\left( {\frac{{1 - w_{j} }}{n - 1}} \right)\left( { - \ln \left( {M_{{\varsigma_{j} }} } \right)} \right)^{\beth } } \right)^{{\frac{1}{\beth }}} }} , \hfill \\ e^{{ - \left( {\left( {\frac{{1 - w_{j} }}{n - 1}} \right)\left( { - \ln \left( {I_{{\varsigma_{j} }} } \right)} \right)^{\beth } } \right)^{{\frac{1}{\beth }}} }} , \hfill \\ \sqrt {1 - e^{{ - \left( {\left( {\frac{{1 - w_{j} }}{n - 1}} \right)\left( { - \ln \left( {1 - \left( {N_{{\varsigma_{j} }} } \right)^{2} } \right)} \right)^{\beth } } \right)^{{\frac{1}{\beth }}} }} } \hfill \\ \end{gathered} \right)$$

$$\mathop {\mathop \otimes \limits_{j = 1} }\limits^{\nu } {\upbeta }_{{\varsigma_{j} }}^{{\frac{{1 - w_{\varsigma } }}{n - 1}}} = \left( \begin{gathered} e^{{ - \left( {\mathop \sum \limits_{j = 1}^{\nu } \left( {\left( {\left( {\frac{{1 - w_{j} }}{n - 1}} \right)\left( { - \ln \left( {M_{{\varsigma_{j} }} } \right)} \right)^{\beth } } \right)} \right)} \right)^{{\frac{1}{\beth }}} }} , \hfill \\ e^{{ - \left( {\mathop \sum \limits_{j = 1}^{\nu } \left( {\left( {\left( {\frac{{1 - w_{j} }}{n - 1}} \right)\left( { - \ln \left( {I_{{\varsigma_{j} }} } \right)} \right)^{\beth } } \right)} \right)} \right)^{{\frac{1}{\beth }}} }} , \hfill \\ \sqrt {1 - e^{{ - \left( {\mathop \sum \limits_{j = 1}^{\nu } \left( {\left( {\frac{{1 - w_{j} }}{n - 1}} \right)\left( { - \ln \left( {1 - \left( {N_{{\varsigma_{j} }} } \right)^{2} } \right)} \right)^{\beth } } \right)} \right)^{{\frac{1}{\beth }}} }} } \hfill \\ \end{gathered} \right)$$

## Spherical fuzzy Aczel-Alsina dual Hammy mean operators

### Definition 13

Let ${\beta }_{1}=\left({M}_{1}, {I}_{1}, {N}_{1}\right)$, ${\beta }_{2}=\left({M}_{2}, {I}_{2}, {N}_{2}\right)$, …, and ${\beta }_{n}=\left({M}_{n}, {I}_{n}, {N}_{n}\right)$ be SFNs. The proposed SFAADHM operator of the SFNs ${\beta }_{1}$, ${\beta }_{2}$, …, and ${\beta }_{n}$ is defined as follows:

9 $$SFAADHM^{\left( \nu \right)} \left( {\beta_{1} ,\beta_{2} , \ldots ,\beta_{n} } \right) = \left( {\mathop \otimes \limits_{{1 \le \varsigma_{1} < , \ldots , < \varsigma_{\nu } \le n}} \frac{{\left( {\mathop {\mathop \oplus \limits_{j = 1} }\limits^{\nu } \beta_{{\varsigma_{j} }} } \right)}}{\nu }} \right)^{{\frac{1}{{C_{n}^{\nu } }}}}$$

where $$1\le \nu \le n$$ and $${C}_{n}^{\nu }=\frac{n!}{\nu !\left(n-\nu \right)!}$$.

### Theorem 6

Let ${\beta }_{1}=\left({M}_{1}, {I}_{1}, {N}_{1}\right)$, ${\beta }_{2}=\left({M}_{2}, {I}_{2}, {N}_{2}\right)$, …, and ${\beta }_{n}=\left({M}_{n}, {I}_{n}, {N}_{n}\right)$ be SFNs. The aggregated value of the SFAADHM operator is a SFN, shown as follows:

10 $$SFAADHM^{\left( \nu \right)} \left( {\beta_{1} ,\beta_{2} , \ldots ,\beta_{n} } \right) = \left( \begin{gathered} e^{{ - \left( {\left( {\frac{1}{{C_{n}^{\nu } }}} \right)\left( {\mathop \sum \limits_{{1 \le \varsigma_{1} < , \ldots , < \varsigma_{\nu } \le n}} \left( { - \ln \left( {\sqrt {1 - e^{{ - \left( {\left( {\frac{1}{\nu }} \right)\left( {\mathop \sum \limits_{j = 1}^{\nu } \left( { - \ln \left( {1 - \left( {M_{{\varsigma_{j} }} } \right)^{2} } \right)} \right)^{\beth } } \right)} \right)^{{\frac{1}{\beth }}} }} } } \right)} \right)^{\beth } } \right)} \right)^{{\frac{1}{\beth }}} }} , \hfill \\ e^{{ - \left( {\left( {\frac{1}{{C_{n}^{\nu } }}} \right)\left( {\mathop \sum \limits_{{1 \le \varsigma_{1} < , \ldots , < \varsigma_{\nu } \le n}} \left( {\left( {\frac{1}{\nu }} \right)\left( {\mathop \sum \limits_{j = 1}^{\nu } \left( { - \ln \left( {I_{{\varsigma_{j} }} } \right)} \right)^{\beth } } \right)} \right)} \right)} \right)^{{\frac{1}{\beth }}} }} , \hfill \\ \sqrt {1 - e^{{ - \left( {\left( {\frac{1}{{C_{n}^{\nu } }}} \right)\left( {\mathop \sum \limits_{{1 \le \varsigma_{1} < , \ldots , < \varsigma_{\nu } \le n}} \left( { - \ln \left( {1 - \left( {e^{{ - \left( {\left( {\frac{1}{\nu }} \right)\left( {\mathop \sum \limits_{j = 1}^{\nu } \left( { - \ln \left( {N_{{\varsigma_{j} }} } \right)} \right)^{\beth } } \right)} \right)^{{\frac{1}{\beth }}} }} } \right)^{2} } \right)} \right)^{\beth } } \right)} \right)^{{\frac{1}{\beth }}} }} } \hfill \\ \end{gathered} \right)$$

### Proof

Is straightforward.

$$\left( {\mathop {\mathop \oplus \limits_{j = 1} }\limits^{\nu } \beta_{{\varsigma_{j} }} } \right) = \left( \begin{gathered} \sqrt {1 - e^{{ - \left( {\mathop \sum \limits_{j = 1}^{\nu } \left( { - \ln \left( {1 - \left( {M_{{\varsigma_{j} }} } \right)^{2} } \right)} \right)^{\beth } } \right)^{{\frac{1}{\beth }}} }} } , \hfill \\ e^{{ - \left( {\mathop \sum \limits_{j = 1}^{\nu } \left( { - \ln \left( {I_{{\varsigma_{j} }} } \right)} \right)^{\beth } } \right)^{{\frac{1}{\beth }}} }} , \hfill \\ e^{{ - \left( {\mathop \sum \limits_{j = 1}^{\nu } \left( { - \ln \left( {N_{{\varsigma_{j} }} } \right)} \right)^{\beth } } \right)^{{\frac{1}{\beth }}} }} \hfill \\ \end{gathered} \right)$$

$$\frac{{\left( {\mathop {\mathop \oplus \limits_{j = 1} }\limits^{\nu } \beta_{{\varsigma_{j} }} } \right)}}{\nu } = \left( \begin{gathered} \sqrt {1 - e^{{ - \left( {\left( {\frac{1}{\nu }} \right)\left( {\mathop \sum \limits_{j = 1}^{\nu } \left( { - \ln \left( {1 - \left( {M_{{\varsigma_{j} }} } \right)^{2} } \right)} \right)^{\beth } } \right)} \right)^{{\frac{1}{\beth }}} }} } , \hfill \\ e^{{ - \left( {\left( {\frac{1}{\nu }} \right)\left( {\mathop \sum \limits_{j = 1}^{\nu } \left( { - \ln \left( {I_{{\varsigma_{j} }} } \right)} \right)^{\beth } } \right)} \right)^{{\frac{1}{\beth }}} }} , \hfill \\ e^{{ - \left( {\left( {\frac{1}{\nu }} \right)\left( {\mathop \sum \limits_{j = 1}^{\nu } \left( { - \ln \left( {N_{{\varsigma_{j} }} } \right)} \right)^{\beth } } \right)} \right)^{{\frac{1}{\beth }}} }} \hfill \\ \end{gathered} \right)$$

$$\mathop \otimes \limits_{{1 \le \varsigma_{1} < , \ldots , < \varsigma_{\nu } \le n}} \frac{{\left( {\mathop {\mathop \oplus \limits_{j = 1} }\limits^{\nu } \beta_{{\varsigma_{j} }} } \right)}}{\nu } = \left( \begin{gathered} e^{{ - \left( {\mathop \sum \limits_{{1 \le \varsigma_{1} < , \ldots , < \varsigma_{\nu } \le n}} \left( { - \ln \left( {\sqrt {1 - e^{{ - \left( {\left( {\frac{1}{\nu }} \right)\left( {\mathop \sum \limits_{j = 1}^{\nu } \left( { - \ln \left( {1 - \left( {M_{{\varsigma_{j} }} } \right)^{2} } \right)} \right)^{\beth } } \right)} \right)^{{\frac{1}{\beth }}} }} } } \right)} \right)^{\beth } } \right)^{{\frac{1}{\beth }}} }} , \hfill \\ e^{{ - \left( {\mathop \sum \limits_{{1 \le \varsigma_{1} < , \ldots , < \varsigma_{\nu } \le n}} \left( {\left( {\frac{1}{\nu }} \right)\left( {\mathop \sum \limits_{j = 1}^{\nu } \left( { - \ln \left( {I_{{\varsigma_{j} }} } \right)} \right)^{\beth } } \right)} \right)} \right)^{{\frac{1}{\beth }}} }} , \hfill \\ \sqrt {1 - e^{{ - \left( {\mathop \sum \limits_{{1 \le \varsigma_{1} < , \ldots , < \varsigma_{\nu } \le n}} \left( { - \ln \left( {1 - \left( {e^{{ - \left( {\left( {\frac{1}{\nu }} \right)\left( {\mathop \sum \limits_{j = 1}^{\nu } \left( { - \ln \left( {N_{{\varsigma_{j} }} } \right)} \right)^{\beth } } \right)} \right)^{{\frac{1}{\beth }}} }} } \right)^{2} } \right)} \right)^{\beth } } \right)^{{\frac{1}{\beth }}} }} } \hfill \\ \end{gathered} \right)$$

$$\left( {\mathop \otimes \limits_{{1 \le \varsigma_{1} < , \ldots , < \varsigma_{\nu } \le n}} \frac{{\left( {\mathop {\mathop \oplus \limits_{j = 1} }\limits^{\nu } \beta_{{\varsigma_{j} }} } \right)}}{\nu }} \right)^{{\frac{1}{{C_{n}^{\nu } }}}} = \left( \begin{gathered} e^{{ - \left( {\left( {\frac{1}{{C_{n}^{\nu } }}} \right)\left( {\mathop \sum \limits_{{1 \le \varsigma_{1} < , \ldots , < \varsigma_{\nu } \le n}} \left( { - \ln \left( {\sqrt {1 - e^{{ - \left( {\left( {\frac{1}{\nu }} \right)\left( {\mathop \sum \limits_{j = 1}^{\nu } \left( { - \ln \left( {1 - \left( {M_{{\varsigma_{j} }} } \right)^{2} } \right)} \right)^{\beth } } \right)} \right)^{{\frac{1}{\beth }}} }} } } \right)} \right)^{\beth } } \right)} \right)^{{\frac{1}{\beth }}} }} , \hfill \\ e^{{ - \left( {\left( {\frac{1}{{C_{n}^{\nu } }}} \right)\left( {\mathop \sum \limits_{{1 \le \varsigma_{1} < , \ldots , < \varsigma_{\nu } \le n}} \left( {\left( {\frac{1}{\nu }} \right)\left( {\mathop \sum \limits_{j = 1}^{\nu } \left( { - \ln \left( {I_{{\varsigma_{j} }} } \right)} \right)^{\beth } } \right)} \right)} \right)} \right)^{{\frac{1}{\beth }}} }} , \hfill \\ \sqrt {1 - e^{{ - \left( {\left( {\frac{1}{{C_{n}^{\nu } }}} \right)\left( {\mathop \sum \limits_{{1 \le \varsigma_{1} < , \ldots , < \varsigma_{\nu } \le n}} \left( { - \ln \left( {1 - \left( {e^{{ - \left( {\left( {\frac{1}{\nu }} \right)\left( {\mathop \sum \limits_{j = 1}^{\nu } \left( { - \ln \left( {N_{{\varsigma_{j} }} } \right)} \right)^{\beth } } \right)} \right)^{{\frac{1}{\beth }}} }} } \right)^{2} } \right)} \right)^{\beth } } \right)} \right)^{{\frac{1}{\beth }}} }} } \hfill \\ \end{gathered} \right)$$

### Definition 14

Let ${\beta }_{1}=\left({M}_{1}, {I}_{1}, {N}_{1}\right)$, ${\beta }_{2}=\left({M}_{2}, {I}_{2}, {N}_{2}\right)$, …, and ${\beta }_{n}=\left({M}_{n}, {I}_{n}, {N}_{n}\right)$ be SFNs. The proposed SFAAWDHM operator ${SFAAWDHM}^{\left(\nu \right)}$ of the SFNs ${\beta }_{1}$, ${\beta }_{2}$, …, and ${\beta }_{n}$ is defined as follows:

11 $$\begin{gathered} SFAAWDHM^{\left( \nu \right)} \left( {\beta_{1} ,\beta_{2} , \ldots ,\beta_{n} } \right) \hfill \\ \quad = \left\{ \begin{gathered} \left( {\mathop \otimes \limits_{{1 \le \varsigma_{1} < \varsigma_{2} < , \ldots , < \varsigma_{\nu } < n }} \left( {1 - \mathop \sum \limits_{j = 1}^{\nu } w_{{\varsigma_{j} }} } \right)\left( {\frac{{\mathop {\mathop \oplus \limits_{j = 1} }\limits^{\nu } \beta_{{\varsigma_{j} }} }}{\nu }} \right)} \right)^{{\frac{1}{{C_{n - 1}^{\nu } }}}} \quad \;1 \le \nu < n \hfill \\ \mathop {\mathop \oplus \limits_{j = 1} }\limits^{\nu } \beta_{\varsigma }^{{\frac{{1 - w_{j} }}{n - 1}}} \quad \quad \quad \quad \quad \quad \quad \quad \quad \quad \quad \quad \quad \quad \quad \quad \quad \quad \quad \quad \,\,\nu = n \hfill \\ \end{gathered} \right. \hfill \\ \end{gathered}$$

where $w_{j}$ be the weight vectors of $\beta_{\varsigma }$ such that $w_{j} \in \left[ {0, 1} \right]$ and $\sum\nolimits_{j = 1}^{n} {w_{j} = 1}$.

### Theorem 7

Let ${\beta }_{1}=\left({M}_{1}, {I}_{1}, {N}_{1}\right)$, ${\beta }_{2}=\left({M}_{2}, {I}_{2}, {N}_{2}\right)$, …, and ${\beta }_{n}=\left({M}_{n}, {I}_{n}, {N}_{n}\right)$ be SFNs. The aggregated result ${SFAAWHM}^{\left(\nu \right)}\left({\beta }_{1},{\beta }_{2},\ldots ,{\beta }_{n}\right)$ of the SFNs ${\beta }_{1}$, ${\beta }_{2}$, …, and ${\beta }_{n}$ is still a SFN, where,

12 $$\begin{gathered} SFAAWDHM^{\left( \nu \right)} \left( {\beta_{1} ,\beta_{2} , \ldots ,\beta_{n} } \right) \hfill \\ \quad = \left\{ \begin{gathered} \left( \begin{gathered} e^{{ - \left( {\left( {\frac{1}{{C_{n - 1}^{\nu } }}} \right)\left( {\mathop \sum \limits_{{1 \le \varsigma_{1} < \varsigma_{2} < , \ldots , < \varsigma_{\nu } < n}} \left( { - \ln \left( {\sqrt {1 - e^{{ - \left( {\left( {1 - \mathop \sum \limits_{j = 1}^{\nu } w_{j} } \right)\left( {\left( {\frac{1}{\nu }} \right)\left( {\mathop \sum \limits_{j = 1}^{\nu } \left( { - \ln \left( {1 - \left( {M_{{\varsigma_{j} }} } \right)^{2} } \right)} \right)^{\beth } } \right)} \right)} \right)^{{\frac{1}{\beth }}} }} } } \right)} \right)^{\beth } } \right)} \right)^{{\frac{1}{\beth }}} }} , \hfill \\ e^{{ - \left( {\left( {\frac{1}{{C_{n - 1}^{\nu } }}} \right)\left( {\mathop \sum \limits_{{1 \le \varsigma_{1} < \varsigma_{2} < , \ldots , < \varsigma_{\nu } < n}} \left( {\left( {1 - \mathop \sum \limits_{j = 1}^{\nu } w_{j} } \right)\left( {\left( {\frac{1}{\nu }} \right)\left( {\mathop \sum \limits_{j = 1}^{\nu } \left( { - \ln \left( {I_{{\varsigma_{j} }} } \right)} \right)^{\beth } } \right)} \right)} \right)} \right)} \right)^{{\frac{1}{\beth }}} }} , \hfill \\ \sqrt {1 - e^{{ - \left( {\left( {\frac{1}{{C_{n - 1}^{\nu } }}} \right)\left( {\mathop \sum \limits_{{1 \le \varsigma_{1} < \varsigma_{2} < , \ldots , < \varsigma_{\nu } < n}} \left( { - \ln \left( {1 - \left( {e^{{ - \left( {\left( {1 - \mathop \sum \limits_{j = 1}^{\nu } w_{j} } \right)\left( {\left( {\frac{1}{\nu }} \right)\left( {\mathop \sum \limits_{j = 1}^{\nu } \left( { - \ln \left( {N_{{\varsigma_{j} }} } \right)} \right)^{\beth } } \right)} \right)} \right)^{{\frac{1}{\beth }}} }} } \right)^{2} } \right)} \right)^{\beth } } \right)} \right)^{{\frac{1}{\beth }}} }} } \hfill \\ \end{gathered} \right)\quad \;1 \le \nu < n \hfill \\ \mathop {\mathop \oplus \limits_{j = 1} }\limits^{\nu } \beta_{{\varsigma_{j} }}^{{\frac{{1 - w_{j} }}{n - 1}}} = \left( \begin{gathered} \sqrt {1 - e^{{ - \left( {\mathop \sum \limits_{j = 1}^{\nu } \left( { - \ln \left( {1 - \left( {e^{{ - \left( {\left( {\frac{{1 - w_{j} }}{n - 1}} \right)\left( { - \ln \left( {M_{{\varsigma_{j} }} } \right)} \right)^{\beth } } \right)^{{\frac{1}{\beth }}} }} } \right)^{2} } \right)} \right)^{\beth } } \right)^{{\frac{1}{\beth }}} }} } , \hfill \\ e^{{ - \left( {\mathop \sum \limits_{j = 1}^{\nu } \left( {\left( {\frac{{1 - w_{j} }}{n - 1}} \right)\left( { - \ln \left( {I_{{\varsigma_{j} }} } \right)} \right)^{\beth } } \right)} \right)^{{\frac{1}{\beth }}} }} , \hfill \\ e^{{ - \left( {\mathop \sum \limits_{j = 1}^{\nu } \left( { - \ln \left( {\sqrt {1 - e^{{ - \left( {\left( {\frac{{1 - w_{j} }}{n - 1}} \right)\left( { - \ln \left( {1 - \left( {N_{{\varsigma_{j} }} } \right)^{2} } \right)} \right)^{\beth } } \right)^{{\frac{1}{\beth }}} }} } } \right)} \right)^{\beth } } \right)^{{\frac{1}{\beth }}} }} \hfill \\ \end{gathered} \right)\quad \quad \quad \quad \quad \quad \quad \quad \quad \quad \quad \nu = n \hfill \\ \end{gathered} \right. \hfill \\ \end{gathered}$$

### Proof

We can prove the above theoream easily.

$$\mathop {\mathop \oplus \limits_{j = 1} }\limits^{\nu } {\upbeta }_{{\varsigma_{j} }} = \left( \begin{gathered} \sqrt {1 - e^{{ - \left( {\mathop \sum \limits_{j = 1}^{\nu } \left( { - \ln \left( {1 - \left( {M_{{\varsigma_{j} }} } \right)^{2} } \right)} \right)^{\beth } } \right)^{{\frac{1}{\beth }}} }} } , \hfill \\ e^{{ - \left( {\mathop \sum \limits_{j = 1}^{\nu } \left( { - \ln \left( {I_{{\varsigma_{j} }} } \right)} \right)^{\beth } } \right)^{{\frac{1}{\beth }}} }} , \hfill \\ e^{{ - \left( {\mathop \sum \limits_{j = 1}^{\nu } \left( { - \ln \left( {N_{{\varsigma_{j} }} } \right)} \right)^{\beth } } \right)^{{\frac{1}{\beth }}} }} \hfill \\ \end{gathered} \right)$$

$$\left( {\frac{{\mathop {\mathop \oplus \limits_{j = 1} }\limits^{\nu } {\upbeta }_{{\varsigma_{j} }} }}{\nu }} \right) = \left( \begin{gathered} \sqrt {1 - e^{{ - \left( {\left( {\frac{1}{\nu }} \right)\left( {\mathop \sum \limits_{j = 1}^{\nu } \left( { - \ln \left( {1 - \left( {M_{{\varsigma_{j} }} } \right)^{2} } \right)} \right)^{\beth } } \right)} \right)^{{\frac{1}{\beth }}} }} } , \hfill \\ e^{{ - \left( {\left( {\frac{1}{\nu }} \right)\left( {\mathop \sum \limits_{j = 1}^{\nu } \left( { - \ln \left( {I_{{\varsigma_{j} }} } \right)} \right)^{\beth } } \right)} \right)^{{\frac{1}{\beth }}} }} , \hfill \\ e^{{ - \left( {\left( {\frac{1}{\nu }} \right)\left( {\mathop \sum \limits_{j = 1}^{\nu } \left( { - \ln \left( {N_{{\varsigma_{j} }} } \right)} \right)^{\beth } } \right)} \right)^{{\frac{1}{\beth }}} }} \hfill \\ \end{gathered} \right)$$

$$\left( {1 - \mathop \sum \limits_{j = 1}^{\nu } w_{j} } \right)\left( {\frac{{\mathop {\mathop \oplus \limits_{j = 1} }\limits^{\nu } {\upbeta }_{{\varsigma_{j} }} }}{\nu }} \right) = \left( \begin{gathered} \sqrt {1 - e^{{ - \left( {\left( {1 - \mathop \sum \limits_{j = 1}^{\nu } w_{j} } \right)\left( {\left( {\frac{1}{\nu }} \right)\left( {\mathop \sum \limits_{j = 1}^{\nu } \left( { - \ln \left( {1 - \left( {M_{{\varsigma_{j} }} } \right)^{2} } \right)} \right)^{\beth } } \right)} \right)} \right)^{{\frac{1}{\beth }}} }} } , \hfill \\ e^{{ - \left( {\left( {1 - \mathop \sum \limits_{j = 1}^{\nu } w_{j} } \right)\left( {\left( {\frac{1}{\nu }} \right)\left( {\mathop \sum \limits_{j = 1}^{\nu } \left( { - \ln \left( {I_{{\varsigma_{j} }} } \right)} \right)^{\beth } } \right)} \right)} \right)^{{\frac{1}{\beth }}} }} , \hfill \\ e^{{ - \left( {\left( {1 - \mathop \sum \limits_{j = 1}^{\nu } w_{j} } \right)\left( {\left( {\frac{1}{\nu }} \right)\left( {\mathop \sum \limits_{j = 1}^{\nu } \left( { - \ln \left( {N_{{\varsigma_{j} }} } \right)} \right)^{\beth } } \right)} \right)} \right)^{{\frac{1}{\beth }}} }} \hfill \\ \end{gathered} \right)$$

$$\begin{gathered} \mathop \otimes \limits_{{1 \le \varsigma_{1} < \varsigma_{2} < , \ldots , < \varsigma_{\nu } < n }} \left( {1 - \mathop \sum \limits_{j = 1}^{\nu } w_{{\varsigma_{j} }} } \right)\left( {\frac{{\mathop {\mathop \oplus \limits_{j = 1} }\limits^{\nu } {\upbeta }_{{\varsigma_{j} }} }}{\nu }} \right) \hfill \\ \quad = \left( \begin{gathered} e^{{ - \left( {\mathop \sum \limits_{{1 \le \varsigma_{1} < \varsigma_{2} < , \ldots , < \varsigma_{\nu } < n}} \left( { - \ln \left( {\sqrt {1 - e^{{ - \left( {\left( {1 - \mathop \sum \limits_{j = 1}^{\nu } w_{j} } \right)\left( {\left( {\frac{1}{\nu }} \right)\left( {\mathop \sum \limits_{j = 1}^{\nu } \left( { - \ln \left( {1 - \left( {M_{{\varsigma_{j} }} } \right)^{2} } \right)} \right)^{\beth } } \right)} \right)} \right)^{{\frac{1}{\beth }}} }} } } \right)} \right)^{\beth } } \right)^{{\frac{1}{\beth }}} }} , \hfill \\ e^{{ - \left( {\mathop \sum \limits_{{1 \le \varsigma_{1} < \varsigma_{2} < , \ldots , < \varsigma_{\nu } < n}} \left( {\left( {1 - \mathop \sum \limits_{j = 1}^{\nu } w_{j} } \right)\left( {\left( {\frac{1}{\nu }} \right)\left( {\mathop \sum \limits_{j = 1}^{\nu } \left( { - \ln \left( {I_{{\varsigma_{j} }} } \right)} \right)^{\beth } } \right)} \right)} \right)} \right)^{{\frac{1}{\beth }}} }} , \hfill \\ \sqrt {1 - e^{{ - \left( {\mathop \sum \limits_{{1 \le \varsigma_{1} < \varsigma_{2} < , \ldots , < \varsigma_{\nu } < n}} \left( { - \ln \left( {1 - \left( {e^{{ - \left( {\left( {1 - \mathop \sum \limits_{j = 1}^{\nu } w_{j} } \right)\left( {\left( {\frac{1}{\nu }} \right)\left( {\mathop \sum \limits_{j = 1}^{\nu } \left( { - \ln \left( {N_{{\varsigma_{j} }} } \right)} \right)^{\beth } } \right)} \right)} \right)^{{\frac{1}{\beth }}} }} } \right)^{2} } \right)} \right)^{\beth } } \right)^{{\frac{1}{\beth }}} }} } \hfill \\ \end{gathered} \right) \hfill \\ \end{gathered}$$

$$\begin{gathered} \left( {\mathop \otimes \limits_{{1 \le \varsigma_{1} < \varsigma_{2} < , \ldots , < \varsigma_{\nu } < n }} \left( {1 - \mathop \sum \limits_{j = 1}^{\nu } w_{{\varsigma_{j} }} } \right)\left( {\frac{{\mathop {\mathop \oplus \limits_{j = 1} }\limits^{\nu } {\upbeta }_{{\varsigma_{j} }} }}{\nu }} \right)} \right)^{{\frac{1}{{C_{n - 1}^{\nu } }}}} \hfill \\ \quad = \left( \begin{gathered} e^{{ - \left( {\left( {\frac{1}{{C_{n - 1}^{\nu } }}} \right)\left( {\mathop \sum \limits_{{1 \le \varsigma_{1} < \varsigma_{2} < , \ldots , < \varsigma_{\nu } < n}} \left( { - \ln \left( {\sqrt {1 - e^{{ - \left( {\left( {1 - \mathop \sum \limits_{j = 1}^{\nu } w_{j} } \right)\left( {\left( {\frac{1}{\nu }} \right)\left( {\mathop \sum \limits_{j = 1}^{\nu } \left( { - \ln \left( {1 - \left( {M_{{\varsigma_{j} }} } \right)^{2} } \right)} \right)^{\beth } } \right)} \right)} \right)^{{\frac{1}{\beth }}} }} } } \right)} \right)^{\beth } } \right)} \right)^{{\frac{1}{\beth }}} }} , \hfill \\ e^{{ - \left( {\left( {\frac{1}{{C_{n - 1}^{\nu } }}} \right)\left( {\mathop \sum \limits_{{1 \le \varsigma_{1} < \varsigma_{2} < , \ldots , < \varsigma_{\nu } < n}} \left( {\left( {1 - \mathop \sum \limits_{j = 1}^{\nu } w_{j} } \right)\left( {\left( {\frac{1}{\nu }} \right)\left( {\mathop \sum \limits_{j = 1}^{\nu } \left( { - \ln \left( {I_{{\varsigma_{j} }} } \right)} \right)^{\beth } } \right)} \right)} \right)} \right)} \right)^{{\frac{1}{\beth }}} }} , \hfill \\ \sqrt {1 - e^{{ - \left( {\left( {\frac{1}{{C_{n - 1}^{\nu } }}} \right)\left( {\mathop \sum \limits_{{1 \le \varsigma_{1} < \varsigma_{2} < , \ldots , < \varsigma_{\nu } < n}} \left( { - \ln \left( {1 - \left( {e^{{ - \left( {\left( {1 - \mathop \sum \limits_{j = 1}^{\nu } w_{j} } \right)\left( {\left( {\frac{1}{\nu }} \right)\left( {\mathop \sum \limits_{j = 1}^{\nu } \left( { - \ln \left( {N_{{\varsigma_{j} }} } \right)} \right)^{\beth } } \right)} \right)} \right)^{{\frac{1}{\beth }}} }} } \right)^{2} } \right)} \right)^{\beth } } \right)} \right)^{{\frac{1}{\beth }}} }} } \hfill \\ \end{gathered} \right) \hfill \\ \end{gathered}$$

*Case 2*: If $\nu =n,$ then we have:

$${\upbeta }_{{\varsigma_{j} }}^{{\frac{{1 - w_{j} }}{n - 1}}} = \left( \begin{gathered} e^{{ - \left( {\left( {\frac{{1 - w_{j} }}{n - 1}} \right)\left( { - \ln \left( {M_{{\varsigma_{j} }} } \right)} \right)^{\beth } } \right)^{{\frac{1}{\beth }}} }} , \hfill \\ e^{{ - \left( {\left( {\frac{{1 - w_{j} }}{n - 1}} \right)\left( { - \ln \left( {I_{{\varsigma_{j} }} } \right)} \right)^{\beth } } \right)^{{\frac{1}{\beth }}} }} , \hfill \\ \sqrt {1 - e^{{ - \left( {\left( {\frac{{1 - w_{j} }}{n - 1}} \right)\left( { - \ln \left( {1 - \left( {N_{{\varsigma_{j} }} } \right)^{2} } \right)} \right)^{\beth } } \right)^{{\frac{1}{\beth }}} }} } \hfill \\ \end{gathered} \right)$$

$$\mathop {\mathop \oplus \limits_{j = 1} }\limits^{\nu } {\upbeta }_{{\varsigma_{j} }}^{{\frac{{1 - w_{j} }}{n - 1}}} = \left( \begin{gathered} \sqrt {1 - e^{{ - \left( {\mathop \sum \limits_{j = 1}^{\nu } \left( { - \ln \left( {1 - \left( {e^{{ - \left( {\left( {\frac{{1 - w_{j} }}{n - 1}} \right)\left( { - \ln \left( {M_{{\varsigma_{j} }} } \right)} \right)^{\beth } } \right)^{{\frac{1}{\beth }}} }} } \right)^{2} } \right)} \right)^{\beth } } \right)^{{\frac{1}{\beth }}} }} } , \hfill \\ e^{{ - \left( {\mathop \sum \limits_{j = 1}^{\nu } \left( {\left( {\frac{{1 - w_{j} }}{n - 1}} \right)\left( { - \ln \left( {I_{{\varsigma_{j} }} } \right)} \right)^{\beth } } \right)} \right)^{{\frac{1}{\beth }}} }} , \hfill \\ e^{{ - \left( {\mathop \sum \limits_{j = 1}^{\nu } \left( { - \ln \left( {\sqrt {1 - e^{{ - \left( {\left( {\frac{{1 - w_{j} }}{n - 1}} \right)\left( { - \ln \left( {1 - \left( {N_{{\varsigma_{j} }} } \right)^{2} } \right)} \right)^{\beth } } \right)^{{\frac{1}{\beth }}} }} } } \right)} \right)^{\beth } } \right)^{{\frac{1}{\beth }}} }} \hfill \\ \end{gathered} \right)$$

## Decision algorithm for multi-attribute group decision-making problem

In this section, we propose a new multi-attribute group decision-making (MAGDM) method based on spherical fuzzy Aczel-Alsina Hammy mean operators. Let ${\varvec{\Gamma}}=\left\{{{\varvec{\Gamma}}}_{1}, {{\varvec{\Gamma}}}_{2}, \ldots , {{\varvec{\Gamma}}}_{\mathbf{n}}\right\}$ be a set of alternatives and let ${\varvec{\Lambda}}=\left\{{{\varvec{\Lambda}}}_{1}, {{\varvec{\Lambda}}}_{2}, \ldots , {{\varvec{\Lambda}}}_{\sigma }\right\}$ be a set of attributes, where the weight of the attribute ${{\varvec{\Lambda}}}_{q}$ is ${\omega }_{q}$, *q* = 1, 2, …, $\sigma$,${\omega }_{q}>0$ and $\sum_{q=1}^{\sigma }{\omega }_{q}=1$. Let $\mathbf{\rm K}=\left\{{\text{\rm K}}_{1}, {\text{\rm K}}_{2}, \ldots , {\text{\rm K}}_{\tau }\right\}$ be a set of experts, where the weight of each expert ${\text{\rm K}}_{\text{r}}$ is ${\Phi }_{r}$, *r* = 1, 2, …, $\tau$, ${\Phi }_{r}>0$ and $\sum_{r=1}^{\tau }{\Phi }_{r}=1$. Let ${\text{\rm K}}^{r}$ $={\left({D}_{pq}^{r}\right)}_{n\times \sigma }$ be the decision matrix given by the decision maker ${\text{\rm K}}_{r}$, where ${D}_{pq}^{r}$ is a SFN, ${D}_{pq}^{r}=\left({M}_{pq}^{r}, {I}_{pq}^{r}, {N}_{pq}^{r}\right)$, *r* = 1, 2, …, $\tau$, *p* = 1, 2, …, $n$ and *q* = 1, 2, …, $\sigma$. The proposed MAGDM method is now presented as follows:

*Step 1*: The decision-maker arranges expert’s opinions in different decision matrices ${\text{\rm K}}^{r}$ $={\left({D}_{pq}^{r}\right)}_{n\times \sigma }$.

*Step 2*: Convert the decision matrix ${\text{\rm K}}^{r}$ $={\left({D}_{pq}^{r}\right)}_{n\times \sigma }$ into the normalized decision matrix ${\overline{\text{\rm K}} }^{r}$ $=$ ${\left({\overline{D} }_{pq}^{r}\right)}_{n\times \sigma }$, where

$$\overline{D}_{pq}^{r} = \left\{ \begin{gathered} \left( {M_{pq}^{r} , I_{pq}^{r} , N_{pq}^{r} } \right),\quad {\text{if}}\;{{\varvec{\Lambda}}}_{q} \;{\text{is}}\;{\text{a}}\;{\text{benifit}}\;{\text{type}}\;{\text{attribute }} \hfill \\ \left( {N_{pq}^{r} , I_{pq}^{r} , M_{pq}^{r} } \right),\quad {\text{if}}\;{{\varvec{\Lambda}}}_{q} \;{\text{is}}\;{\text{a}}\;{\text{cost}}\;{\text{type}}\;{\text{attribute}} \hfill \\ \end{gathered} \right.$$

*r* = 1, 2, …, $$\tau$$, *p* = 1, 2, …, $$n$$ and *q* = 1, 2, …, $$\sigma$$.

*Step 3*: Calculated results by using the information of given decision matrices under our invented methodologies, including SFAWHM and SFAWDHM operators at $\nu =1$.

*Step 4*: Utilize the consequences obtained from our invented approaches and compute results by using the discussed approaches of SFAWHM and SFAWDHM operators from aggregated information of SFNs. Let $\nu =2$.

*Step 5*: Obtained score function using the following expression:

$$S\left(\beta \right)=\frac{1}{3}\left(2+{M}^{2}\left(\xi \right)-{I}^{2}\left(\xi \right)-{N}^{2}\left(\xi \right)\right)$$

*Step 6*: Assess suitable alternatives by using ranking and ordering techniques based on score values.

### Case study

In the following, we use an example to illustrate the proposed MAGDM method. The automobile industry is booming with an increasing number of countries participating in the production of automobiles, while the market power structure is constantly changing. The automobile industry’s contribution to the potential for expansion is dependent on the inclusion of motorized transportation in the national infrastructure of the economy. Suppose that three experts are going to evaluate five automobile companies ${\nabla }_{1}, {\nabla }_{2}, {\nabla }_{3}, {\nabla }_{4}, {\nabla }_{5}$ based on four attributes ${\Lambda }_{1}$: Reliable Manufacturing Structure, ${\Lambda }_{2}$: price of Fuel Energy Consumption,${\Lambda }_{3}$: Availability of Manufactured Parts, and ${\Lambda }_{4}$: Comfortable and Modern Design. Three experts ${\text{\rm K}}_{1}$, ${\text{\rm K}}_{2}$ and ${\text{\rm K}}_{3}$ are invited to evaluate the five automobile companies ${\nabla }_{1}, {\nabla }_{2}, {\nabla }_{3}, {\nabla }_{4}, {\nabla }_{5}$ with respect to the four attributes ${\Lambda }_{1}$, ${\Lambda }_{2}$, ${\Lambda }_{3}$, ${\Lambda }_{4}$ using SFNs to get the decision matrices ${\text{\rm K}}^{1}$, ${\text{\rm K}}^{2}$ and ${\text{\rm K}}^{3}$, as shown in Tables 2, 3 and 4, respectively, where the weights of the experts ${\text{\rm K}}_{1}$, ${\text{\rm K}}_{2}$ and ${\text{\rm K}}_{3}$ are 0.30, 0.25 and 0.45, respectively, and the weights of the attributes ${\Lambda }_{1}$, ${\Lambda }_{2}$, ${\Lambda }_{3}$, ${\Lambda }_{4}$ are 0.30, 0.15, 0.20 and 0.35, respectively.

**Table 2:** Decision matrix ${D}^{1}$ given by an expert ${\rm K}_{1}$.

	${\Lambda }_{1}$	${\Lambda }_{2}$	${\Lambda }_{3}$	${\Lambda }_{4}$
${\nabla }_{1}$	$\left(0.61, 0.45, 0.14\right)$	$\left(0.22, 0.54, 0.70\right)$	$\left(0.76, 0.13, 0.32\right)$	$\left(0.56, 0.65, 0.22\right)$
${\nabla }_{2}$	$\left(0.38, 0.63, 0.34\right)$	$\left(0.61, 0.10, 0.45\right)$	$\left(0.70, 0.60, 0.21\right)$	$\left(0.23, 0.34, 0.56\right)$
${\nabla }_{3}$	$\left(0.16, 0.43, 0.32\right)$	$\left(0.12, 0.32, 0.56\right)$	$\left(0.45, 0.65, 0.39\right)$	$\left(0.60, 0.20, 0.54\right)$
${\nabla }_{4}$	$\left(0.56, 0.18, 0.56\right)$	$\left(0.65, 0.29, 0.34\right)$	$\left(0.70, 0.29, 0.34\right)$	$\left(0.61, 0.51, 0.41\right)$
${\nabla }_{5}$	$\left(0.45, 0.81, 0.15\right)$	$\left(0.67, 0.51, 0.21\right)$	$\left(0.23, 0.34, 0.56\right)$	$\left(0.43, 0.81, 0.23\right)$

**Table 3:** Decision matrix ${D}^{2}$ given by an expert ${\rm K}_{2}$.

	${\Lambda }_{1}$	${\Lambda }_{2}$	${\Lambda }_{3}$	${\Lambda }_{4}$
${\nabla }_{1}$	$\left(0.61, 0.51, 0.41\right)$	$\left(0.23, 0.43, 0.56\right)$	$\left(0.21, 0.45, 0.78\right)$	$\left(0.54, 0.21, 0.39\right)$
${\nabla }_{2}$	$\left(0.22, 0.44, 0.67\right)$	$\left(0.56, 0.22, 0.77\right)$	$\left(0.76, 0.45, 0.23\right)$	$\left(0.88, 0.22, 0.19\right)$
${\nabla }_{3}$	$\left(0.21, 0.67, 0.11\right)$	$\left(0.56, 0.43, 0.11\right)$	$\left(0.67, 0.54, 0.29\right)$	$\left(0.41, 0.34, 0.51\right)$
${\nabla }_{4}$	$\left(0.78, 0.28, 0.43\right)$	$\left(0.56, 0.55, 0.43\right)$	$\left(0.78, 0.11, 0.45\right)$	$\left(0.27, 0.70, 0.60\right)$
${\nabla }_{5}$	$\left(0.65, 0.21, 0.45\right)$	$\left(0.56, 0.34, 0.67\right)$	$\left(0.34, 0.50, 0.51\right)$	$\left(0.77, 0.11, 0.34\right)$

**Table 4:** Decision matrix ${D}^{3}$ given by an expert ${\rm K}_{3}$.

	${\Lambda }_{1}$	${\Lambda }_{2}$	${\Lambda }_{3}$	${\Lambda }_{4}$
${\nabla }_{1}$	$\left(0.71, 0.51, 0.21\right)$	$\left(0.21, 0.56, 0.76\right)$	$\left(0.60, 0.39, 0.23\right)$	$\left(0.72, 0.32, 0.12\right)$
${\nabla }_{2}$	$\left(0.31, 0.65, 0.67\right)$	$\left(0.71, 0.56, 0.22\right)$	$\left(0.29, 0.59, 0.66\right)$	$\left(0.21, 0.30, 0.20\right)$
${\nabla }_{3}$	$\left(0.23, 0.56, 0.73\right)$	$\left(0.55, 0.29, 0.31\right)$	$\left(0.44, 0.76, 0.22\right)$	$\left(0.70, 0.50, 0.10\right)$
${\nabla }_{4}$	$\left(0.45, 0.62, 0.48\right)$	$\left(0.60, 0.19, 0.29\right)$	$\left(0.48, 0.12, 0.71\right)$	$\left(0.55, 0.21, 0.19\right)$
${\nabla }_{5}$	$\left(0.71, 0.61, 0.10\right)$	$\left(0.39, 0.41, 0.31\right)$	$\left(0.62, 0.54, 0.11\right)$	$\left(0.18, 0.49, 0.56\right)$

In the following, the proposed MAGDM method is applied to deal with this example, shown as follows:

*Step 1*: Decision-makers collect information about four criteria associated with each alternative and list them in different decision matrices of Tables 2, 3 and 4.

*Step 2*: We applied our discussed methodologies to evaluate the information on the given decision matrices for $\nu =\beth =1$. The results obtained by the SFAWHM and SFAWDHM operators are shown in Tables 5 and 6, respectively.

**Table 5:** Reveals the consequences of the SFAWHM operator at $\nu =\beth =1$.

	${\Lambda }_{1}$	${\Lambda }_{2}$	${\Lambda }_{3}$	${\Lambda }_{4}$
${\nabla }_{1}$	$\left(0.6415, 0.4881, 0.2342\right)$	$\left(0.2212, 0.5008, 0.6585\right)$	$\left(0.5973, 0.2801, 0.4072\right)$	$\left(0.6082, 0.3502, 0.2308\right)$
${\nabla }_{2}$	$\left(0.3175, 0.5655, 0.5108\right)$	$\left(0.6279, 0.2075, 0.4401\right)$	$\left(0.6607, 0.5439, 0.2964\right)$	$\left(0.6359, 0.2852, 0.2969\right)$
${\nabla }_{3}$	$\left(0.1980, 0.5341, 0.2837\right)$	$\left(0.4508, 0.3428, 0.2804\right)$	$\left(0.5391, 0.6389, 0.3026\right)$	$\left(0.5876, 0.3057, 0.3334\right)$
${\nabla }_{4}$	$\left(0.6356, 0.2920, 0.4926\right)$	$\left(0.6096, 0.3179, 0.3513\right)$	$\left(0.6898, 0.1660, 0.4560\right)$	$\left(0.5162, 0.4429, 0.3756\right)$
${\nabla }_{5}$	$\left(0.6082, 0.4831, 0.1917\right)$	$\left(0.5777, 0.4210, 0.3408\right)$	$\left(0.4210, 0.4377, 0.3472\right)$	$\left(0.5640, 0.3687, 0.3336\right)$

**Table 6:** Reveals the consequences of the SFAWDHM operator $\nu =\beth =1$.

	${\Lambda }_{1}$	${\Lambda }_{2}$	${\Lambda }_{3}$	${\Lambda }_{4}$
${\nabla }_{1}$	$\left(0.4024, 0.5268, 0.2474\right)$	$\left(0.0760, 0.5576, 0.7561\right)$	$\left(0.2393, 0.3485, 0.5536\right)$	$\left(0.3635, 0.4592, 0.2247\right)$
${\nabla }_{2}$	$\left(0.1191, 0.6403, 0.6552\right)$	$\left(0.3833, 0.3711, 0.5895\right)$	$\left(0.3030, 0.6015, 0.4644\right)$	$\left(0.1595, 0.2795, 0.3339\right)$
${\nabla }_{3}$	$\left(0.0647, 0.6235, 0.5189\right)$	$\left(0.1456, 0.3554, 0.3573\right)$	$\left(0.2776, 0.7326, 0.2894\right)$	$\left(0.3199, 0.3904, 0.4198\right)$
${\nabla }_{4}$	$\left(0.3432, 0.4437, 0.5277\right)$	$\left(0.3600, 0.3749, 0.3618\right)$	$\left(0.3996, 0.1432, 0.5918\right)$	$\left(0.2276, 0.5520, 0.4438\right)$
${\nabla }_{5}$	$\left(0.3551, 0.6771, 0.2477\right)$	$\left(0.2907, 0.4425, 0.4806\right)$	$\left(0.1663, 0.5098, 0.4330\right)$	$\left(0.1861, 0.6169, 0.4328\right)$

*Step 3*: Our discussed numerical example has single types of attributes, so there is no need to perform the second step of the algorithm.

*Step 4*: Compute results by the SFAWHM and SFAWDHM operators by utilizing the revealed consequences shown in Tables 5 and 6 for parametric values of $\nu =2, \beth =1,$ and the obtained results are shown in Table 7.

**Table 7:** Consequences of SFAWHM and SFAWDHM operators.

SFAWHM	SFAWDHM
$\left(0.5008, 0.4134, 0.4174\right)$	$\left(0.0047, 0.8537, 0.8160\right)$
$\left(0.5553, 0.4208, 0.4004\right)$	$\left(0.0044, 0.8488, 0.8727\right)$
$\left(0.4293, 0.4722, 0.2977\right)$	$\left(0.0031, 0.8810, 0.8039\right)$
$\left(0.6185, 0.3039, 0.4227\right)$	$\left(0.0083, 0.7835, 0.8590\right)$
$\left(0.5438, 0.4323, 0.3059\right)$	$\left(0.0049, 0.9028, 0.8005\right)$

*Step 5*: To choose the best optimal option, we compute score values by using the outcomes of SFAWHM and SFAWDHM operators shown in Table 7. All obtained results of score values are shown in Table 8.

**Table 8:** Shows the score values obtained by the SFAWHM and SFAWDHM operators.

Operators	$S\left({\nabla }_{1}\right)$	$S\left({\nabla }_{2}\right)$	$S\left({\nabla }_{3}\right)$	$S\left({\nabla }_{4}\right)$	$S\left({\nabla }_{5}\right)$	Ordering and Ranking
SFAWHM	$0.6352$	$0.6570$	$0.6242$	$0.7039$	$0.6717$	${\nabla }_{4}\succ {\nabla }_{5}\succ {\nabla }_{2}\succ {\nabla }_{1}\succ {\nabla }_{3}$
SFAWDHM	$0.2018$	$0.1726$	$0.1926$	$0.2161$	$0.1814$	${\nabla }_{4}\succ {\nabla }_{1}\succ {\nabla }_{3}\succ {\nabla }_{5}\succ {\nabla }_{2}$

**Fig. 2:**
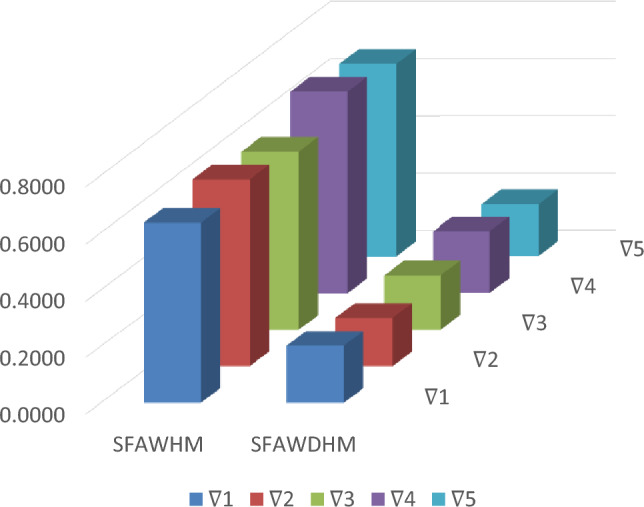
The score values of the alternatives obtained by the proposed SFAWHM operator and the proposed SFAWDHM operator, respectively.

*Step 6*: Evaluate the optimal automobile company by ranking the score values of both proposed methodologies of SFAWHM and SFAWDHM operators. All obtained score values of SFAWHM and SFAWDHM operators are shown graphically in Fig. 2.

## Influence study

**Fig. 3:**
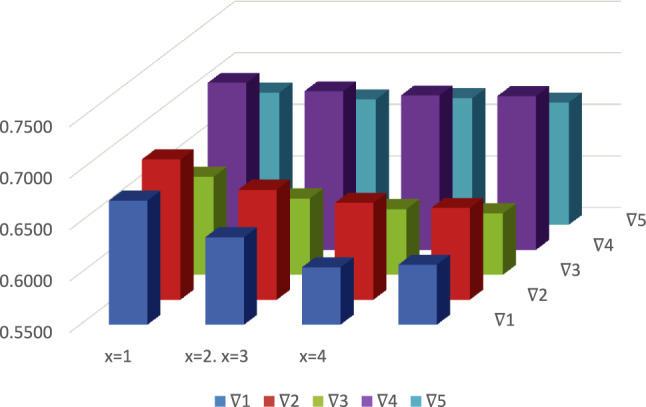
Score values for different parametric values of $\nu_{\varsigma }$ obtained by the SFAWHM operator.

**Fig. 4:**
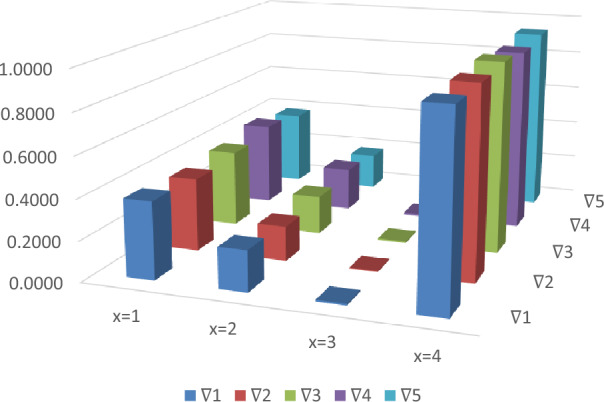
Score values for different parametric values of $\nu_{\varsigma }$ obtained by the SFAWDHM operator.

We analyze different parametric values of $\nu , which$ play a vital role in the process of aggregating information by our proposed methodologies. We computed different results of score value by assigning some different parametric values of $\nu$ and studied the ranking of calculated results by SFAWHM and SFAWDHM operators. All results obtained by SFAWHM and SFAWDHM operators are shown in Tables 9 and 10, respectively. Table 9 shows more details of the obtained results by setting different parametric values of $\nu ,$ and we observed that the ranking of computed results is the same when the degree of parametric values gradually increases. This shows the reliability of our invented approach to the SFAWHM operator. Similarly, Table 10 reveals the consequences of SFAWDHM operators by assigning some different parametric degrees of $\nu$. We also studied the behavior of computed results and the ranking of score values obtained by SFAWDHM operators, which are depicted in Table 10. We see that when parametric degrees of $\nu$ change, the obtained results of score value are different, but the ranking of score values remains the same. These characteristics of the results obtained show the effectiveness and flexibility of our invented methodologies. Further studies of computed results are shown in Tables 9 and 10 in graphical representations of Figs. 3 and 4, respectively.

**Table 9:** Shows the score values obtained by the SFAWHM for the different values of ${x}_{i}$.

Operators	$S\left({\nabla }_{1}\right)$	$S\left({\nabla }_{2}\right)$	$S\left({\nabla }_{3}\right)$	$S\left({\nabla }_{4}\right)$	$S\left({\nabla }_{5}\right)$	Ordering and Ranking
$x=1$	$0.6707$	$0.6864$	$0.6452$	$0.7122$	$0.6783$	${\nabla }_{4}\succ {\nabla }_{5}\succ {\nabla }_{1}\succ {\nabla }_{2}\succ {\nabla }_{3}$
$x=2$	$0.6352$	$0.6570$	$0.6242$	$0.7039$	$0.6717$	${\nabla }_{4}\succ {\nabla }_{5}\succ {\nabla }_{2}\succ {\nabla }_{1}\succ {\nabla }_{3}$
$x=3$	$0.6058$	$0.6442$	$0.6140$	$0.6997$	$0.6729$	${\nabla }_{4}\succ {\nabla }_{5}\succ {\nabla }_{2}\succ {\nabla }_{3}\succ {\nabla }_{1}$
$x=4$	$0.6715$	$0.7024$	$0.6659$	$0.7558$	$0.7259$	${\nabla }_{4}\succ {\nabla }_{5}\succ {\nabla }_{2}\succ {\nabla }_{3}\succ {\nabla }_{1}$

**Table 10:** Shows the score values obtained by the SFAWDHM for the different values of $\nu_{\varsigma }$.

Operators	$S\left({\nabla }_{1}\right)$	$S\left({\nabla }_{2}\right)$	$S\left({\nabla }_{3}\right)$	$S\left({\nabla }_{4}\right)$	$S\left({\nabla }_{5}\right)$	Ordering and Ranking
$x=1$	$0.3819$	$0.3626$	$0.3827$	$0.4188$	$0.3811$	${\nabla }_{4}\succ {\nabla }_{3}\succ {\nabla }_{1}\succ {\nabla }_{5}\succ {\nabla }_{2}$
$x=2$	$0.2018$	$0.1726$	$0.1926$	$0.2161$	$0.1814$	${\nabla }_{4}\succ {\nabla }_{1}\succ {\nabla }_{3}\succ {\nabla }_{5}\succ {\nabla }_{2}$
$x=3$	$0.0079$	$0.0052$	$0.0072$	$0.0101$	$0.0066$	${\nabla }_{4}\succ {\nabla }_{1}\succ {\nabla }_{3}\succ {\nabla }_{5}\succ {\nabla }_{2}$
$x=4$	$0.9363$	$0.9384$	$0.9495$	$0.9139$	$0.9357$	${\nabla }_{3}\succ {\nabla }_{2}\succ {\nabla }_{5}\succ {\nabla }_{1}\succ {\nabla }_{4}$

The following Table 11 demonstrates the ranking of preferences by the setting of the parametric variable in the proposed Aczel Alsina mathematical models of the SFAWHM and SFAWDHM operators. From Table 11, we can analyze that the ranking of preferences remains constant at the parametric variable of Aczel Alsina operators $\beth \ge 15$. This consistency of the ranking of alternatives shows the efficiency and reliability of the proposed mathematical models and decision analysis techniques.

**Table 11:** Explores the result of the ranking of preferences by changing different parametric variables in Aczel Alsina aggregation operators.

Parametric variable	Ranking of alternatives by the SFAWHM operator at $x=2$	Ranking of alternatives by the SFAWDHM operator at $x=2$
$\beth =1$	${\nabla }_{4}\succ {\nabla }_{5}\succ {\nabla }_{2}\succ {\nabla }_{1}\succ {\nabla }_{3}$	${\nabla }_{4}\succ {\nabla }_{1}\succ {\nabla }_{3}\succ {\nabla }_{5}\succ {\nabla }_{2}$
$\beth =3$	${\nabla }_{4}\succ {\nabla }_{5}\succ {\nabla }_{2}\succ {\nabla }_{1}\succ {\nabla }_{3}$	${\nabla }_{4}\succ {\nabla }_{1}\succ {\nabla }_{3}\succ {\nabla }_{5}\succ {\nabla }_{2}$
$\beth =5$	${\nabla }_{4}\succ {\nabla }_{2}\succ {\nabla }_{1}\succ {\nabla }_{5}\succ {\nabla }_{3}$	${\nabla }_{4}\succ {\nabla }_{1}\succ {\nabla }_{3}\succ {\nabla }_{5}\succ {\nabla }_{2}$
$\beth =15$	${\nabla }_{2}\succ {\nabla }_{1}\succ {\nabla }_{4}\succ {\nabla }_{3}\succ {\nabla }_{5}$	${\nabla }_{4}\succ {\nabla }_{3}\succ {\nabla }_{1}\succ {\nabla }_{5}\succ {\nabla }_{2}$
$\beth =25$	${\nabla }_{2}\succ {\nabla }_{1}\succ {\nabla }_{4}\succ {\nabla }_{3}\succ {\nabla }_{5}$	${\nabla }_{4}\succ {\nabla }_{3}\succ {\nabla }_{1}\succ {\nabla }_{5}\succ {\nabla }_{2}$
$\beth =35$	${\nabla }_{2}\succ {\nabla }_{1}\succ {\nabla }_{4}\succ {\nabla }_{3}\succ {\nabla }_{5}$	${\nabla }_{4}\succ {\nabla }_{3}\succ {\nabla }_{1}\succ {\nabla }_{5}\succ {\nabla }_{2}$
$\beth =50$	${\nabla }_{2}\succ {\nabla }_{1}\succ {\nabla }_{4}\succ {\nabla }_{3}\succ {\nabla }_{5}$	${\nabla }_{4}\succ {\nabla }_{3}\succ {\nabla }_{1}\succ {\nabla }_{5}\succ {\nabla }_{2}$
$\beth =65$	${\nabla }_{2}\succ {\nabla }_{1}\succ {\nabla }_{4}\succ {\nabla }_{3}\succ {\nabla }_{5}$	${\nabla }_{4}\succ {\nabla }_{3}\succ {\nabla }_{1}\succ {\nabla }_{5}\succ {\nabla }_{2}$
$\beth =80$	${\nabla }_{2}\succ {\nabla }_{1}\succ {\nabla }_{4}\succ {\nabla }_{3}\succ {\nabla }_{5}$	${\nabla }_{4}\succ {\nabla }_{3}\succ {\nabla }_{1}\succ {\nabla }_{5}\succ {\nabla }_{2}$
$\beth =95$	${\nabla }_{2}\succ {\nabla }_{1}\succ {\nabla }_{4}\succ {\nabla }_{3}\succ {\nabla }_{5}$	${\nabla }_{4}\succ {\nabla }_{3}\succ {\nabla }_{1}\succ {\nabla }_{5}\succ {\nabla }_{2}$
$\beth =100$	${\nabla }_{2}\succ {\nabla }_{1}\succ {\nabla }_{4}\succ {\nabla }_{3}\succ {\nabla }_{5}$	${\nabla }_{4}\succ {\nabla }_{3}\succ {\nabla }_{1}\succ {\nabla }_{5}\succ {\nabla }_{2}$

## Comparatives study

This section demonstrates the superiority and effectiveness of the discussed decision-making methodologies and mathematical approaches under consideration of the spherical fuzzy framework. To achieve the main goal of the comparison method, we have applied existing decision-making terminologies with developed mathematical aggregation operators by different research scholars. For instance, Güner and Aygün^10^ proposed a decision-making approach for handling a group of expert opinions using theoretical concepts of Einstein aggregation operators. We applied the proposed terminologies^10^ to the given expert’s opinions, which are listed in Tables 2, 3 and 4. After integrating the expert’s judgments and the comprehensive aggregation process, we have investigated the ranking of preferences listed in Table 12.

**Table 12:** Reveals a comparative study.

Methodologies	Ranking of preferences
SFAWHM (Current work)	${\nabla }_{4}\succ {\nabla }_{3}\succ {\nabla }_{1}\succ {\nabla }_{5}\succ {\nabla }_{2}$
SFAWDHM (Current work)	${\nabla }_{4}\succ {\nabla }_{3}\succ {\nabla }_{1}\succ {\nabla }_{5}\succ {\nabla }_{2}$
GSFEWA by Güner and Aygün^10^	${\nabla }_{4}\succ {\nabla }_{1}\succ {\nabla }_{5}\succ {\nabla }_{3}\succ {\nabla }_{2}$
GSFEWG by Güner and Aygün^10^	${\nabla }_{1}\succ {\nabla }_{4}\succ {\nabla }_{5}\succ {\nabla }_{2}\succ {\nabla }_{3}$
SFAAWA by Naeem and Ali^45^	${\nabla }_{1}\succ {\nabla }_{4}\succ {\nabla }_{5}\succ {\nabla }_{3}\succ {\nabla }_{2}$
SFAAWG by Naeem and Ali^45^	${\nabla }_{1}\succ {\nabla }_{5}\succ {\nabla }_{4}\succ {\nabla }_{2}\succ {\nabla }_{3}$
SFWA by Ashraf and Abdullah^56^	${\nabla }_{1}\succ {\nabla }_{5}\succ {\nabla }_{4}\succ {\nabla }_{2}\succ {\nabla }_{3}$
SFWG by Ashraf and Abdullah^56^	${\nabla }_{4}\succ {\nabla }_{1}\succ {\nabla }_{5}\succ {\nabla }_{3}\succ {\nabla }_{2}$
Ahmed et al.^57^	Limited structure
Farid and Riaz^58^	Limited structure
Ali and Yang^59^	Limited structure

Furthermore, Naeem and Ali^45^ modified the decision-making approach for handling the group exporter’s judgments with robust mathematical approaches of Aczel Alsina aggregation operators. After applying aggregational terminologies and stepwise decision algorithms discussed in^45^, we examined the ranking of alternatives that are listed in Table 12. We also checked the supremacy of derived decision-making terminologies with another reliable existing approach^56^. Ashraf and Abdullah^56^ also established dominant decision-making methodologies for handling groups of decision-makers by incorporating spherical fuzzy situations. The ranking of preferences is investigated by applying mathematical methodologies^56^ and is listed in Table 12.

**Fig. 5:**
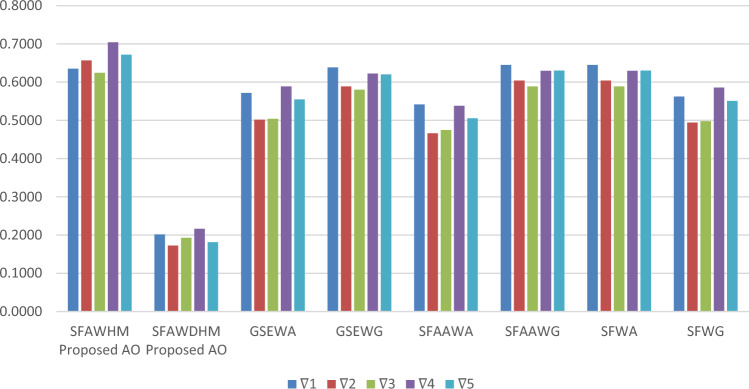
Shows the results of existing mathematical approaches. *Abbreviations*: Generlized spherical fuzzy Einstein weighted average (GSFEWA), generlized spherical fuzzy Einstein weighted geometric (GSFEWG), spherical fuzzy Aczel Alsina weighted average (GSFAAWA), spherical fuzzy Aczel Alsina weighted geometric (SFAAWG), spherical fuzzy weighted average (SFWA), spherical fuzzy weighted geometric (SFWG).

Some existing mathematical appraoches^57–59^ unable to integarate human judgments listed in Tables 2, 3 and 4 due to limited and narrow struture of fuzzy framework. However, Table 12 expores the ranking of prefernces obtanied by different previous mathematical approaches and decision analysis sytem. The proposed Aczel–Alsina Hamy mean operators and their associated decision algorithms for the MAGDM problems are considered superior to many other mathematical operators because they provide a more flexible and robust framework for aggregating complex and uncertain information from multiple experts. The traditional operators may be overly rigid or sensitive to extreme values, the Aczel–Alsina Hamy mean incorporates adjustable parameters that capture non-linear interactions among attributes, effectively balancing the influence of high and low values. This adaptability allows decision-makers to model realistic group dynamics, handle hesitation and vagueness, and reduce bias in consensus formation (Fig. 5).

### Implication of proposed methodologies

The proposed methodologies of the spherical fuzzy framework and Aczél–Alsina Hamy mean models hold strong implications across a wide range of real-life applications where uncertainty, vagueness, and group preferences play a crucial role. In renewable energy selection, they allow policymakers to evaluate multiple energy alternatives by incorporating diverse expert judgments while managing ambiguity in performance indicators. In healthcare decision-making, these models can effectively aggregate doctors’ or specialists’ opinions for disease diagnosis, treatment planning, or prioritizing medical resources, ensuring fair and reliable outcomes. In supply chain and risk management, they help organizations handle uncertain demand, supplier evaluations, and risk factors through structured aggregation of multiple viewpoints. Similarly, in policy planning and smart city development, the methodologies support government bodies in synthesizing heterogeneous expert inputs to design sustainable, inclusive strategies. Moreover, in financial decision-making, they provide robust tools for portfolio selection, credit evaluation, and investment strategies under uncertainty. The overall implication lies in their ability to ensure fairness, adaptability, and reliability, thereby improving the quality of complex group decision-making processes in dynamic real-world scenarios.

## Conclusions

MAGDM is an essential component of current cognitive technologies and has seen significant recent development in the disciplines of management, marketing, and other professions. Effective and powerful models of HM and DHM tools are also studied; these models are used to explore the interrelationship among different arguments. In this article, we generalized the theory of SF information and its fundamental operations. Aczel Alsina triangular norms are more efficient aggregation tools to cope with awkward and inherent fuzzy information. Keeping inspiration from HM, DHM models, and Aczel Alsina triangular tools, we explored the system of SFSs and developed a list of new AOs, including SFAHM and SFAWHM operators. Moreover, we also established certain AOs of SFADHM and SFADWHM operators based on effective tools of Aczel Alsina triangular norms. Some characteristics of our proposed methodologies are also discussed. A MAGDM methodology is utilized to solve a particular numerical example for the selection process of the automobile under our invented methodologies. A comprehensive discussion also presents a study of the effects of different parametric values on the results of our proposed AOs. The proposed MAGDM approach has several advantages over existing approaches, including the capability to handle scenarios in which the input arguments are SFNs and consider the relationships between multi-input arguments, as well as the capability to prevent the arbitrary membership value of some alternative is zero. Additionally, the suggested approach has significant applicability and dependability and may be used to solve a variety of real-world decision-making issues, including identifying a pilot facility, controlling health care, selecting a supplier, and deciding in an urgent situation.

## Limitations and drawbacks

While the proposed methodologies of Aczel Alsina Hamy mean operators and decision-making models provide significant advantages in handling uncertainty and aggregating expert judgments, they also come with certain limitations and drawbacks. First, the computational complexity of these models increases as the number of attributes and alternatives, and making large-scale decision problems time-consuming. Second, the data requirement is relatively high, as experts must provide detailed membership, non-membership, and hesitancy information, which may cause fatigue or inconsistencies in responses. Additionally, the lack of standardization in applying these methodologies across different domains may hinder the comparability and reproducibility of results. Overall, despite their strong theoretical foundations, these methods face challenges in terms of computational efficiency, practical interpretability, and broader real-world adoption.

## Future directions

In the future, our aim is to explore currently discussed robust mathematical approaches and decision-making models to different fuzzy frameworks such as bipolar fuzzy soft sets, complex bipolar fuzzy soft sets, linear Diophantine fuzzy frameworks, bipolar complex fuzzy sets and proportional hesitant fuzzy environments. Furthermore, interesting direction for future research is to modify the mathematical approaches of Aczel Alsina Hamy operators and decision analysis approaches such as Maclaurin symmetric mean operators, EDAS method, MARCOS method and VIKOR method. However, many real-life applications may also be resolved by applying diagnosed mathematical approaches and decision analysis systems such as healthcare systems, renewable energy, transportation problems and risk analysis in investment plans.

## Data Availability

All data generated or analyzed during this study are included in this published article.

## References

[1] Atanasov, K. T. Intuitionistic fuzzy sets. *Fuzzy Sets Syst.* **20**, 87–96 (1986).

[2] Zadeh, L. A. Fuzzy sets. *Inf. Control* **8**(3), 338–353. 10.1016/S0019-9958(65)90241-X (1965).

[3] Yager, R. R. Pythagorean fuzzy subsets. In *2013 Joint IFSA World Congress and NAFIPS Annual Meeting (IFSA/NAFIPS)* 57–61. 10.1109/IFSA-NAFIPS.2013.6608375 (2013).

[4] Yager, R. R. Generalized orthopair fuzzy sets. *IEEE Trans. Fuzzy Syst.* **25**(5), 1222–1230. 10.1109/TFUZZ.2016.2604005 (2017).

[5] Cuong, B. C. Picture fuzzy sets-first results. Part 1, seminar neuro-fuzzy systems with applications (Institute of Mathematics, 2013).

[6] Cuong, B. Picture fuzzy sets. *J. Comput. Sci. Cybern.* **30**, 409. 10.15625/1813-9663/30/4/5032 (2015).

[7] Mahmood, T., Ullah, K., Khan, Q. & Jan, Q. An approach toward decision-making and medical diagnosis problems using the concept of spherical fuzzy sets. *Neural Comput. Appl.* **31**(11), 7041–7053 (2019).

[8] Cuong, B. C. Pythagorean picture fuzzy sets, part 1-basic notions. *J. Comput. Sci. Cybern.* **35**(4), 293–304 (2019).

[9] Ayaz, T., Al-Shomrani, M. M., Abdullah, S. & Hussain, A. Evaluation of enterprise production based on spherical cubic Hamacher aggregation operators. *Mathematics* **8**(10), 1761. 10.3390/math8101761 (2020).

[10] Güner, E. & Aygün, H. Generalized spherical fuzzy Einstein aggregation operators: Application to multi-criteria group decision-making problems. In *Conference Proceedings of Science and Technology* 227–235 (2020).

[11] Xu, Z. Intuitionistic fuzzy aggregation operators. *IEEE Trans. Fuzzy Syst.* **15**(6), 1179–1187 (2007).

[12] Xu, Z. Some similarity measures of intuitionistic fuzzy sets and their applications to multiple attribute decision making. *Fuzzy Optim. Decis. Mak.* **6**(2), 109–121 (2007).

[13] Arora, R. & Garg, H. Group decision-making method based on prioritized linguistic intuitionistic fuzzy aggregation operators and its fundamental properties. *Comp. Appl. Math.* **38**(2), 36. 10.1007/s40314-019-0764-1 (2019).

[14] Chen, X., Suo, C. & Li, Y. Distance measures on intuitionistic hesitant fuzzy set and its application in decision-making. *Comput. Appl. Math.* **40**(3), 1–21 (2021).

[15] Garg, H. A novel correlation coefficients between Pythagorean fuzzy sets and its applications to decision-making processes. *Int. J. Intell. Syst.* **31**(12), 1234–1252. 10.1002/int.21827 (2016).

[16] Akram, M., Dudek, W. A. & Dar, J. M. Pythagorean Dombi fuzzy aggregation operators with application in multicriteria decision-making. *Int. J. Intell. Syst.* **34**(11), 3000–3019 (2019).

[17] Ali, Z., Mahmood, T., Ullah, K. & Khan, Q. Einstein geometric aggregation operators using a novel complex interval-valued Pythagorean fuzzy setting with application in green supplier chain management. *Rep. Mech. Eng.* **2**(1), 105–134. 10.31181/rme2001020105t (2021).

[18] Mahmood, T., Waqas, H. M., Ali, Z., Ullah, K. & Pamucar, D. Frank aggregation operators and analytic hierarchy process based on interval-valued picture fuzzy sets and their applications. *Int. J. Intell. Syst.* **36**(12), 7925–7962 (2021).

[19] Ahmed, D. & Dai, B. Picture fuzzy rough set and rough picture fuzzy set on two different universes and their applications. *J. Math.* **2020**, e8823580. 10.1155/2020/8823580 (2020).

[20] Hussain, A., Ullah, K., Pamucar, D. & Vranješ, Đ. A multi-attribute decision-making approach for the analysis of vendor management using novel complex picture fuzzy Hamy mean operators. *Electronics* **11**(23), 3841 (2022).

[21] Jana, C., Senapati, T., Pal, M. & Yager, R. R. Picture fuzzy Dombi aggregation operators: Application to MADM process. *Appl. Soft Comput.* **74**, 99–109. 10.1016/j.asoc.2018.10.021 (2019).

[22] Akram, M., Khan, A. & Karaaslan, F. Complex spherical Dombi fuzzy aggregation operators for decision-making. *J. Mult. Valued Logic Soft Comput.* **37**, 503–531 (2021).

[23] Ashraf, S., Abdullah, S. & Mahmood, T. Spherical fuzzy Dombi aggregation operators and their application in group decision making problems. *J. Ambient. Intell. Humaniz. Comput.* **11**(7), 2731–2749 (2020).

[24] Fahmi, A., Amin, F., Abdullah, S. & Ali, A. Cubic fuzzy Einstein aggregation operators and its application to decision-making. *Int. J. Syst. Sci.* **49**(11), 2385–2397. 10.1080/00207721.2018.1503356 (2018).

[25] Hussain, A., Ullah, K., Ali, Z., Moslem, S. & Senapati, T. An enhanced physical education evaluation algorithm for higher education using interval valued fermatean fuzzy information. *Soc. Econ. Plan. Sci.* **101**, 102280. 10.1016/j.seps.2025.102280 (2025).

[26] Garg, H. Generalized Pythagorean fuzzy geometric aggregation operators using Einstein t-norm and t-conorm for multicriteria decision-making process. *Int. J. Intell. Syst.* **32**(6), 597–630 (2017).

[27] Gao, H., Wei, G. & Huang, Y. Dual hesitant bipolar fuzzy Hamacher prioritized aggregation operators in multiple attribute decision making. *IEEE Access* **6**, 11508–11522 (2017).

[28] Wu, S.-J. & Wei, G.-W. Pythagorean fuzzy Hamacher aggregation operators and their application to multiple attribute decision making. *Int. J. Knowl. Based Intell. Eng. Syst.* **21**(3), 189–201 (2017).

[29] Darko, A. P. & Liang, D. Some q-rung orthopair fuzzy Hamacher aggregation operators and their application to multiple attribute group decision making with modified EDAS method. *Eng. Appl. Artif. Intell.* **87**, 103259 (2020).

[30] Li, D., Wan, G. & Rong, Y. An enhanced spherical cubic fuzzy WASPAS method and its application for the assessment of service quality of crowdsourcing logistics platform. *Spectr. Decis. Mak. Appl.* **3**(1), 100–123 (2026).

[31] Hussain, A., Ullah, K., Pamucar, D. & Simic, V. Intuitionistic fuzzy Sugeno-Weber decision framework for sustainable digital security assessment. *Eng. Appl. Artif. Intell.* **137**, 109085 (2024).

[32] Imran, R., Ullah, K., Ali, Z. & Akram, M. A multi-criteria group decision-making approach for robot selection using interval-valued intuitionistic fuzzy information and Aczel-Alsina Bonferroni means. *Spectr. Decis. Mak. Appl.* **1**(1), 1–32 (2024).

[33] Asif, M., Ishtiaq, U. & Argyros, I. K. Hamacher aggregation operators for pythagorean fuzzy set and its application in multi-attribute decision-making problem. *Spectr. Oper. Res.* **2**(1), 27–40 (2025).

[34] Fujita, T. Shadowed offset: Integrating offset and shadowed set frameworks for enhanced uncertainty modelling. *Spectr. Oper. Res.* 1–17 (2025).

[35] Shit, C. & Ghorai, G. Charging method selection of a public charging station using an interval-valued picture fuzzy bidirectional projection based on vikor method with unknown attribute weights. *Information* **16**(2), 94 (2025).

[36] Shit, C. & Ghorai, G. A novel Aczel-Alsina aggregation operators based multi-criteria group decision making approach under Hesitant fuzzy set for the selection of best brand in Educational Institution. *Eng. Appl. Artif. Intell.* **158**, 111464 (2025).

[37] Shit, C. & Ghorai, G. Multiple attribute decision-making based on different types of Dombi aggregation operators under Fermatean fuzzy information. *Soft Comput.* **25**(22), 13869–13880. 10.1007/s00500-021-06252-9 (2021).

[38] Shit, C., Ghorai, G., Xin, Q. & Gulzar, M. Harmonic aggregation operator with trapezoidal picture fuzzy numbers and its application in a multiple-attribute decision-making problem. *Symmetry* **14**(1), 135 (2022).

[39] Senapati, T., Chen, G. & Yager, R. R. Aczel–Alsina aggregation operators and their application to intuitionistic fuzzy multiple attribute decision making. *Int. J. Intell. Syst.* **37**(2), 1529–1551 (2022).

[40] Hussain, A. et al. Enhancing renewable energy evaluation: Utilizing complex picture fuzzy frank aggregation operators in multi-attribute group decision-making. *Sustain. Cities Soc.* **116**, 105842 (2024).

[41] Senapati, T., Chen, G., Mesiar, R. & Yager, R. R. Novel Aczel-Alsina operations-based interval-valued intuitionistic fuzzy aggregation operators and their applications in multiple attribute decision-making process. *Int. J. Intell. Syst.* **37**(8), 5059–5081 (2021).

[42] Hussain, A., Ullah, K., Alshahrani, M. N., Yang, M.-S. & Pamucar, D. Novel Aczel-Alsina operators for pythagorean fuzzy sets with application in multi-attribute decision making. *Symmetry* **14**(5), 940 (2022).

[43] Naeem, M., Khan, Y., Ashraf, S., Weera, W. & Batool, B. A novel picture fuzzy Aczel-Alsina geometric aggregation information: Application to determining the factors affecting mango crops. *AIMS Math.* **7**(7), 12264–12288 (2022).

[44] Hussain, A., Ullah, K., Zhang, J. & Mahmood, T. Intuitionistic fuzzy muirhead means motivated by frank triangular norms. *Comput. Appl. Math.* **43**(6), 320. 10.1007/s40314-024-02661-2 (2024).

[45] Naeem, M. & Ali, J. A novel multi-criteria group decision-making method based on Aczel-Alsina spherical fuzzy aggregation operators: Application to evaluation of solar energy cells. *Phys. Scr.* **97**(8), 085203 (2022).

[46] Hara, T., Uchiyama, M. & Takahasi, S.-E. A refinement of various mean inequalities. *J. Inequal. Appl.* **1998**(4), 932025 (1998).

[47] Wu, L., Wang, J. & Gao, H. Models for competiveness evaluation of tourist destination with some interval-valued intuitionistic fuzzy Hamy mean operators. *J. Intell. Fuzzy Syst.* **36**(6), 5693–5709. 10.3233/JIFS-181545 (2019).

[48] Wu, L., Wei, G., Gao, H. & Wei, Y. Some interval-valued intuitionistic fuzzy Dombi Hamy mean operators and their application for evaluating the elderly tourism service quality in tourism destination. *Mathematics* **6**(12), 294. 10.3390/math6120294 (2018).

[49] Liu, P. & Liu, X. Linguistic intuitionistic fuzzy hamy mean operators and their application to multiple-attribute group decision making. *IEEE Access* **7**, 127728–127744 (2019).

[50] Li, Z., Wei, G. & Lu, M. Pythagorean fuzzy Hamy mean operators in multiple attribute group decision making and their application to supplier selection. *Symmetry* **10**(10), 505. 10.3390/sym10100505 (2018).

[51] Qin, J. Interval type-2 fuzzy Hamy mean operators and their application in multiple criteria decision making. *Granul. Comput.* **2**(4), 249–269. 10.1007/s41066-017-0041-x (2017).

[52] Liu, P. & Wang, Y. Intuitionistic fuzzy interaction Hamy mean operators and their application to multi-attribute group decision making. *Group Decis. Negot.* **28**(1), 197–232. 10.1007/s10726-018-9601-y (2019).

[53] Wang, J. et al. Some q-rung orthopair fuzzy Hamy mean operators in multiple attribute decision-making and their application to enterprise resource planning systems selection. *Int. J. Intell. Syst.* **34**(10), 2429–2458 (2019).

[54] Kutlu Gundogdu, F. & Kahraman, C. Extension of WASPAS with spherical fuzzy sets. *Informatica* **30**(2), 269–292 (2019).

[55] Aczél, J. & Alsina, C. Characterizations of some classes of quasilinear functions with applications to triangular norms and to synthesizing judgements. *Aeq. Math.* **25**(1), 313–315. 10.1007/BF02189626 (1982).

[56] Ashraf, S. & Abdullah, S. Spherical aggregation operators and their application in multiattribute group decision-making. *Int. J. Intell. Syst.* **34**(3), 493–523. 10.1002/int.22062 (2019).

[57] Ahmed, M., Ashraf, S. & Mashat, D. S. Complex intuitionistic hesitant fuzzy aggregation information and their application in decision making problems. *ATAMS* **2**(1), 1–21. 10.56578/atams020101 (2024).

[58] Farid, H. M. A. & Riaz, M. Linear diophantine fuzzy information aggregation with multi-criteria decision-making. In *Fuzzy Optimization, Decision-making and Operations Research* (eds Jana, C. et al.) 281–317 (Springer International Publishing, 2023).

[59] Ali, Z. & Yang, M.-S. Analysis of renewable energies based on circular bipolar complex intuitionistic Fuzzy Linguistic information with frank power aggregation operators and MABAC model. *Int. J. Comput. Intell. Syst.* **18**(1), 82. 10.1007/s44196-025-00800-z (2025).

